# Identification of a neuronal population in the telencephalon essential for fear conditioning in zebrafish

**DOI:** 10.1186/s12915-018-0502-y

**Published:** 2018-04-25

**Authors:** Pradeep Lal, Hideyuki Tanabe, Maximiliano L. Suster, Deepak Ailani, Yuri Kotani, Akira Muto, Mari Itoh, Miki Iwasaki, Hironori Wada, Emre Yaksi, Koichi Kawakami

**Affiliations:** 1 Division of Molecular and Developmental Biology, National Institute of Genetics, Mishima, Shizuoka, 411-8540 Japan; 2 Department of Genetics, SOKENDAI (The Graduate University for Advanced Studies), Mishima, Shizuoka, 411-8540 Japan; 3 Kavli Institute for Systems Neuroscience and Centre for the Biology of Memory, Norwegian Brain Centre, Norwegian University of Science and Technology (NTNU), Trondheim, Norway; 4 Present address: Visual Interaction GmbH, Warthestrasse 21, 14513 Teltow, Germany; 5 Present address: College of Liberal Arts and Sciences, Kitasato University, Sagamihara, Kanagawa 252-0373 Japan

**Keywords:** gene trapping, enhancer trapping, transposable element, fear conditioning, Pavlovian conditioning, botulinum neurotoxin, Gal4-UAS, dorsomedial telencephalon, amygdala

## Abstract

**Background:**

Fear conditioning is a form of learning essential for animal survival and used as a behavioral paradigm to study the mechanisms of learning and memory. In mammals, the amygdala plays a crucial role in fear conditioning. In teleost, the medial zone of the dorsal telencephalon (Dm) has been postulated to be a homolog of the mammalian amygdala by anatomical and ablation studies, showing a role in conditioned avoidance response. However, the neuronal populations required for a conditioned avoidance response via the Dm have not been functionally or genetically defined.

**Results:**

We aimed to identify the neuronal population essential for fear conditioning through a genetic approach in zebrafish. First, we performed large-scale gene trap and enhancer trap screens, and created transgenic fish lines that expressed Gal4FF, an engineered version of the Gal4 transcription activator, in specific regions in the brain. We then crossed these Gal4FF-expressing fish with the effector line carrying the botulinum neurotoxin gene downstream of the Gal4 binding sequence UAS, and analyzed the double transgenic fish for active avoidance fear conditioning. We identified 16 transgenic lines with Gal4FF expression in various brain areas showing reduced performance in avoidance responses. Two of them had Gal4 expression in populations of neurons located in subregions of the Dm, which we named 120A-Dm neurons. Inhibition of the 120A-Dm neurons also caused reduced performance in Pavlovian fear conditioning. The 120A-Dm neurons were mostly glutamatergic and had projections to other brain regions, including the hypothalamus and ventral telencephalon.

**Conclusions:**

Herein, we identified a subpopulation of neurons in the zebrafish Dm essential for fear conditioning. We propose that these are functional equivalents of neurons in the mammalian pallial amygdala, mediating the conditioned stimulus–unconditioned stimulus association. Thus, the study establishes a basis for understanding the evolutionary conservation and diversification of functional neural circuits mediating fear conditioning in vertebrates.

**Electronic supplementary material:**

The online version of this article (10.1186/s12915-018-0502-y) contains supplementary material, which is available to authorized users.

## Background

Fear conditioning is a type of learning through which animals learn to predict an aversive event from a correlated environmental cue. In mammals, the amygdala plays essential roles in this type of learning [1, 2]. The mammalian amygdala is a complex and anatomically heterogeneous structure, consisting of approximately 20 subnuclei that are derivatives of the pallial and subpallial portions of the telencephalon. The pallial amygdala consists of cortical nuclei and basolateral nuclei (BLA), containing predominantly glutamatergic neurons, whereas the subpallial amygdala consists of medial and central nuclei (CeA), containing predominantly GABAergic neurons [3–5]. The roles of these nuclei in fear conditioning have been studied extensively by producing nuclei-specific lesions. The BLA has been shown to serve as the sensory interface essential for the association of a conditioned (CS) and an unconditioned stimulus (US). The CeA then receives inputs from BLA through intra-amygdaloid circuitry and serves as the primary output structure, with projections to other brain regions and controlling fear responses [1, 2, 6, 7].

Fear conditioning is an evolutionarily conserved behavior, and both active avoidance and Pavlovian fear conditioning have been described in teleost fish [8–11]. In teleost, it has been hypothesized that the forebrain is formed by a mechanism called eversion, while the mammalian forebrain is formed by evagination. Additionally, the teleost telencephalon is composed of area dorsalis and area ventralis, which are homologous to the pallium and subpallium and are rich in glutamatergic and GABAergic neurons, respectively [12–15]. From neuroanatomical and hodological studies, it has been proposed that the medial zone of the dorsal telencephalon (Dm) is a homolog of the mammalian amygdala [16, 17]. A functional study on the telencephalic region important for fear conditioning was performed by ablation experiments [18], wherein goldfish were trained in active avoidance fear conditioning and, after acquisition of the conditioned avoidance response, the medial pallium (MP), including the Dm area, was ablated by surgery. The MP-lesioned fish exhibited a deficit in performing the avoidance response, indicating that the MP area was essential for retention of the conditioned avoidance response. Although the functional study supported the hypothesis that the teleost Dm is a homolog of the mammalian amygdala, the lesions created were quite large, and specific neuronal populations or circuits essential for fear conditioning have yet to be explored.

In zebrafish, Lau et al. [19] analyzed *c-fos* expression patterns in the brain when fish performed a light/dark choice (light-avoidance) behavior, and found that *c-fos* expression was detected in cells in Dm and other brain regions, including the hypothalamus and ventral telencephalon. von Trotha et al. [20] also analyzed *c-fos* expression in the zebrafish brain during administration of amphetamine, a drug of abuse, and an amphetamine-induced place preference behavior, and detected *c-fos* expression in the Dm area. Thus, these studies suggest the involvement of neurons in Dm in emotional and motivational behaviors. However, the cells found in these studies are not genetically labeled, and are therefore not manipulatable, and the requirements for behaviors are unknown.

Herein, we aim to identify the neuronal population essential for fear conditioning through a genetic approach in zebrafish. In previous studies [21, 22], we developed transposon-mediated gene trap and enhancer trap methods, and generated transgenic fish lines that expressed Gal4FF, an engineered Gal4 transcription activator, in specific organs, tissues and cells, including specific neuronal populations. Further, we demonstrated that, by taking advantage of the Gal4-UAS binary system, the activity of such specific neurons can be inhibited by targeted expression of the tetanus neurotoxin gene [21, 23]. In this study, we applied this powerful approach to explore the adult brain function. First, we performed large-scale gene trap and enhancer trap screens and identified transgenic fish lines that expressed Gal4FF in various different regions in the adult brain. Second, we selected lines expressing Gal4FF predominantly in the forebrain, crossed them with the UAS-botulinum neurotoxin fish that contained a modified botulinum toxin (BoTx) gene downstream of UAS [24], and analyzed behaviors of the double transgenic fish by using fear conditioning paradigms. Finally, we found transgenic fish lines expressing Gal4FF in a subpopulation of neurons in Dm that showed reduced performance in fear conditioning when crossed with the UAS:BoTx fish. Thus, the present study genetically identified the neuronal population in zebrafish essential for fear conditioning, which may be a functional equivalent of the mammalian amygdala.

## Results

### Identification of transgenic fish with Gal4FF expression in the adult brain

**Fig. 1:**
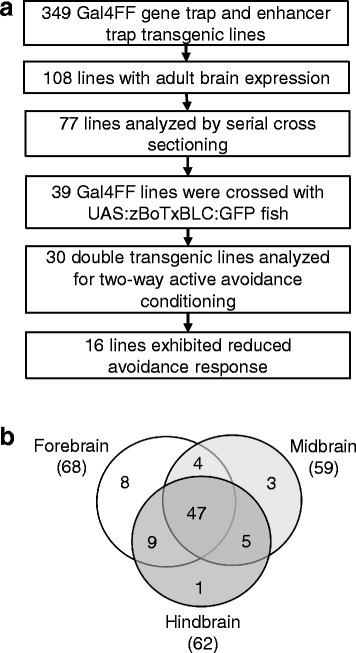
Identification of transgenic zebrafish with Gal4FF expression in the adult brain. **a** Outline of the genetic screen for transgenic fish with deficits in active avoidance fear conditioning. **b** Classification of GFP expression patterns in the selected 77 Gal4FF;UAS:GFP transgenic lines

The outline of this study is shown in Fig. 1a. We performed large-scale genetic screens by using *Tol2* transposon-based gene trap and enhancer trap constructs [21], and generated transgenic fish that expressed Gal4FF, an engineered Gal4 transcription activator, in spatially and temporally restricted fashions at the embryonic stages. From this collection, we selected 349 transgenic fish, including 174 lines with Gal4FF expression in the central nervous system (CNS) and 175 lines without CNS expression at the embryonic stages, crossed them with the UAS:GFP fish, and analyzed the GFP expression patterns at the adult stage. We first observed their brains externally, then opened the skulls, and identified 108 lines with detectable levels of GFP expression in the adult brain. Among those, 93 already had Gal4FF expression in the CNS at embryonic stages (93/174; 53%), whereas 15 did not (15/175; 8.6%). Thus, 93 lines with adult brain expression patterns would have been obtained by screening 174 embryonic CNS lines, suggesting prescreening for CNS expression at embryonic stages should enrich fish with Gal4FF expression in the adult brain. From these 108 lines, we selected 77 lines that showed strong GFP expression, and analyzed them further by making coronal sections. The GFP (Gal4FF) expression was observed in the forebrain (68 lines), midbrain (59 lines), and hindbrain (62 lines) (Fig. 1b).

### Identification of Gal4FF transgenic fish with deficits in two-way active avoidance fear conditioning

**Fig. 2:**
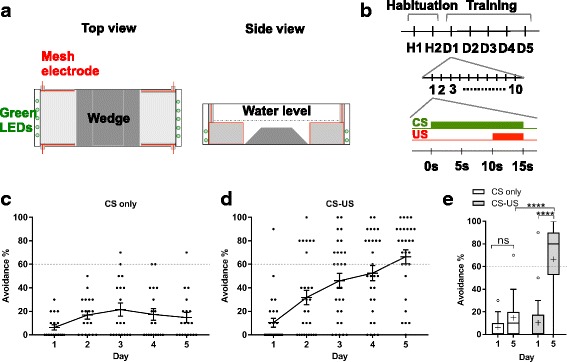
Two-way active avoidance fear conditioning. **a** Shuttle box used for active avoidance conditioning. Top view (left) and side view (right). **b** Scheme for active avoidance conditioning. After habituation for 2 days, fish underwent 10 trials (with 25 ± 5 s intervals) per day over 5 consecutive days. In each trial, conditioned stimulus (CS; green LED) was presented followed by an unconditioned stimulus (US; electric shock) 10 s after CS, for 5 s; after this, both CS and US were turned off. If fish escaped during CS, then US was not given. **c**, **d** Performance of active avoidance response (%) of wild type fish treated only by CS (CS only: *n* = 19) (**c**) and by CS and US (CS-US: *n* = 28) (**d**). **e** Comparison of performance of active avoidance response of fish treated with CS only and with CS-US in Tukey box plot. Outliers are shown in open circles. Mean is marked by ‘+’. Two-way ANOVA, fish groups (CS-US wild type, CS-only wild type, and all double transgenic fish including fish described in Fig. 3 and Additional file 2: Figure S1) × training days (day 1, day 5), was performed (*F* = 7.236, *P* < 0.0001). Dunnett’s multiple comparison post-hoc tests were performed between CS-US and CS-only wild type fish on sessions on day 1 and day 5 (see also Fig. 3 and Additional file 2: Figure S1). The Student’s *t* test was performed on CS-US wild type fish between day 1 and day 5. ****P < 0.0001; ns, not significant


We aimed to identify neuronal populations essential for fear conditioning. For this purpose, we set up an assay system for two-way active avoidance conditioning using a shuttle box with two compartments (Fig. 2a). Green LEDs were used as a CS, and electric shocks given 10 s after CS were used as a US. When fish moved to another compartment prior to the US, which was defined as “escape” or “active avoidance”, the shock was not given. Ten trials per day were conducted on 5 consecutive days (Fig. 2b). In this paradigm, wild type zebrafish showed increased escape responses through the 5 days, compared with fish to which only CS was given (Fig. 2c–e). Thus, active avoidance conditioning using this protocol was efficient and reproducible (Additional file 1: Movie S1).

Additional file 1:**Movie S1.** Active avoidance fear conditioning of wild type fish. The movie shows an example of the analysis. On day 1, wild type fish were placed in a white acrylic tank, and US (electric shock) was given 10 s after CS (green LED) was on. On day 5, the fish successfully escaped to another compartment after CS was on. (MOV 3374 kb)


**Fig. 3:**
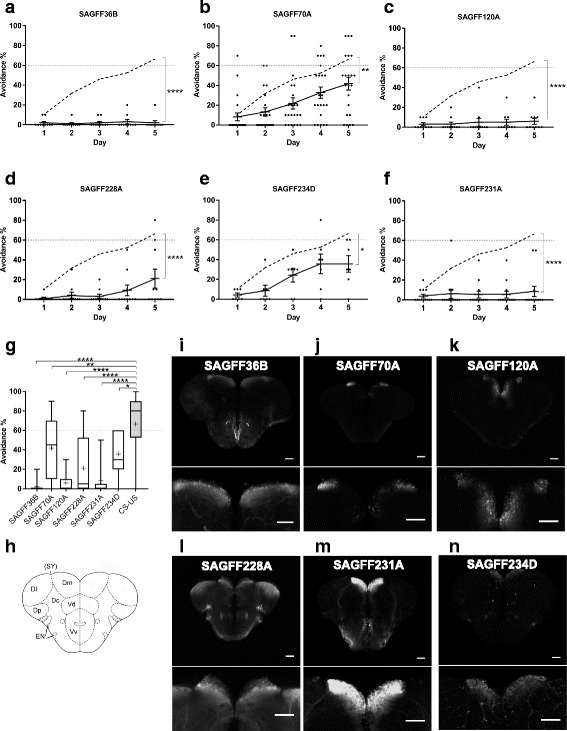
Gal4FF transgenic fish lines that showed deficits in the active avoidance response and had expression patterns in the Dm. **a**–**f** Performance of two-way active avoidance response of wild type fish (*n* = 28) (Fig. 2 and shown in dotted lines) and double transgenic fish that are created by crossing the Gal4FF transgenic fish with the UAS:zBoTxBLC:GFP fish. **a** SAGFF36B (*n* = 10). (**b**) SAGFF70A (*n* = 22). (**c**) SAGFF120A (*n* = 10). **d** SAGFF228A (*n* = 10). **e** SAGFF231A (*n* = 13). **f** SAGFF234D (*n* = 11). Means ± SEM and avoidance (%) for individual fish are plotted. **g** Comparison of performance of avoidance responses at day 5 with Tukey box plot. Mean is marked by ‘+’. Two-way ANOVA, fish groups (CS-US wild type, CS only wild type described in Fig. 2c, d and all double transgenic fish including fish described in Additional file 2: Figure S1) x training days (day 1, day 5), was performed (*F* = 7.236, *P* < 0.0001). Dunnett’s multiple comparison post-hoc tests were performed between wild type and double transgenic fish on day 5. *P < 0.05, **P < 0.01, ***P < 0.001, and ****P < 0.0001. ns, not significant (*P* > 0.05). **h** A coronal view of the zebrafish telencephalon. Dm, medial zone of dorsal telencephalic area (D); Dl, lateral zone of D; Dc, central zone of D; Dp, posterior zone of D; SY, sulcus ypsiloniformis; Vd, dorsal nucleus of ventral telencephalic area (V); Vv, ventral nucleus of V; EN, entopeduncular nucleus. **i**–**n** GFP expression patterns in the coronal section of the Gal4FF;UAS:GFP transgenic fish with magnified views of Dm. The Gal4FF transgenic fish are crossed with UAS:GFP effector fish. **i** SAGFF36B. **j** SAGFF70A. **k** SAGFF120A. **l** SAGFF228A. **m** SAGFF231A. **n** SAGFF234D. Scale bars, 200 μm

In order to inhibit neuronal functions, we constructed a transgenic fish line that carried a codon-optimized botulinum toxin B light chain gene fused to the EGFP sequence downstream of the UAS sequence (UAS:zBoTxBLC:GFP). We have already shown that the UAS:zBoTxBLC:GFP line could efficiently inhibit neuronal activities in combination with Gal4 drivers at the larval stages [24]. From the 77 “adult brain” Gal4FF lines, we selected 39 lines that had rather restricted Gal4 expression in the forebrain and crossed them with the UAS:zBoTxBLC:GFP line. While nine double transgenic lines exhibited lethal locomotion deficits during larval stages, 30 double transgenic lines could survive to adulthood. We then analyzed the 30 Gal4FF;UAS:zBoTxBLC:GFP double transgenic fish by using the two-way active avoidance paradigm; 14 lines performed the avoidance response comparable to wild type fish and 16 lines showed significantly reduced performance in acquisition of the avoidance response (Fig. 3a–f, Additional file 2: Figure S1). We analyzed all of these 16 lines by Southern blot hybridization, and confirmed that the Gal4FF expression patterns were generated by single insertions of either the gene or enhancer trap construct.

**Fig. 4:**
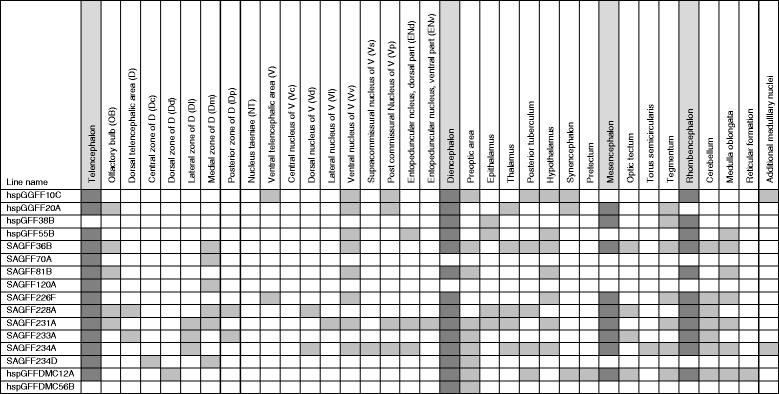
Gal4FF expression patterns in the brain of 16 transgenic lines that showed a deficit in active avoidance fear conditioning. A summary of GFP expression patterns of the Gal4FF transgenic lines (crossed with the UAS:GFP fish) that showed deficits in active avoidance fear conditioning when crossed with the UAS:zBoTxBLC:GFP fish (Additional file 2: Figures S1 and Additional file 3: and Figure S2). The brain are divided into four major regions (telencephalon, diencephalon, mesencephalon, and rhombencephalon), and further subdivided mainly according to Wullimann et al. [59]. Shadowed boxes indicate regions where GFP expression was detected under an epifluorescence microscope

We then analyzed the 16 Gal4FF;UAS:GFP lines by making serial cross sections (Fig. 4, Additional file 3: Figure S2); 14 lines (hspGGFF10C, hspGGFF20A, hspGFF55B, SAGFF36B, SAGFF70A, SAGFF81B, SAGFF120A, SAGFF226F, SAGFF228A, SAGFF231A, SAGFF233A, SAGFF234A, SAGFF234D, and hspGFFDMC12A) showed GFP expression in the telencephalon and other brain regions. In the telencephalon, GFP expression was prominently observed in the medial zone of the dorsal telencephalon (Dm; 6 lines) and in the ventral nucleus of the ventral telencephalon (Vv: 8 lines). The other two lines, hspGFF38B and hspGFFDMC56B, showed strong GFP expression in the diencephalon and mesencephalon, including the habenula (Hb) nuclei and tegmentum, and in the preoptic area in the diencephalon, respectively.

### The *emx3* enhancer trap lines showed deficits in two-way active avoidance fear conditioning when crossed with the UAS-neurotoxin line

Six lines (SAGFF36B, SAGFF70A, SAGFF120A, SAGFF228A, SAGFF231A, and SAGFF234D) exhibited reduced active avoidance responses and showed Gal4FF expression in the Dm area, which has been postulated to be a homolog of the mammalian amygdala (Fig. 3g–n). We analyzed these Dm lines by inverse PCR, and determined the integration sites of the gene trap or enhancer trap construct (Table 1).

**Table 1:** Transposon integration sites in transgenic lines with Gal4FF expression in Dm

Line name	Chromosome	Features	Trapped gene/ nearest gene
SAGFF36B	Chr8	intergenic	*fgf17 / zgc:123194-001*
SAGFF70A	Chr14	intergenic	*emx3*
SAGFF120A	Chr14	intergenic	*emx3*
SAGFF228A^**a**^	ND	ND	repetitive sequence
SAGFF231A	Chr6	intergenic	*si:dkey-166d12.2*
SAGFF234D	Chr5	intron	*orai2*

The transposon integration sites in transgenic lines that had Gal4FF expression in Dm were cloned by inverse PCR, sequenced and mapped on the zebrafish genome. *fgf17*: *fibroblast growth factor 17. emx3*: *empty spiracles homeobox 3. si:dkey-166d12.2: RAP1 GTPase Activating Protein. orai2*: *ORAI calcium release-activated calcium modulator 2*

^**a**^The integration site in SAGFF228A was mapped in a repetitive sequence and the locus could not be determined

**Fig. 5:**
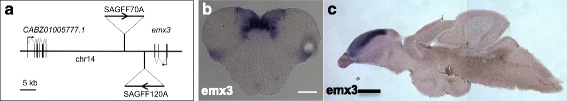
SAGFF70A and SAGFF120A are enhancer trap lines of the *emx3* gene. **a** Transposon integration sites in the SAGFF70A and SAGFF120A transgenic fish. **b**, **c** In situ hybridization analysis of the adult brain using the *emx3* probe. **b** Coronal section, scale bar 200 μm. **c** Sagittal section, scale bar 500 μm

Among these, two lines, SAGFF70A and SAGFF120A, had rather specific Gal4FF (UAS:GFP) expression patterns in the Dm area (Fig. 3j, k). In the SAGFF70A and SAGFF120A fish, the transposon insertions were located on chromosome 14, both near the *emx3* gene (Fig. 5a). The *emx3* gene is expressed in the dorsal telencephalon and part of the diencephalon at larval stages, and in the Dm area at the adult stage [25–27]. We performed in situ hybridization using the *emx3* probe, and confirmed its expression in Dm at the adult stage (Fig. 5b, c). SAGFF70A;UAS:GFP and SAGFF120A;UAS:GFP fish expressed GFP in Dm at the adult stage, and in the dorsal telencephalon and part of the diencephalon at embryonic stages, recapitulating the expression pattern of the *emx3* gene (Additional file 4: Figure S3). These results indicate that, in the SAGFF70A and SAGFF120A lines, Gal4FF is expressed under the control of the *emx3* enhancer(s). GFP expression in Dm was slightly stronger in SAGFF120A;UAS:GFP fish, in which the transposon construct was located closer to the *emx3* gene than in SAGFF70A;UAS:GFP fish. Consistent with this, the deficit observed in active avoidance conditioning was more severe in SAGFF120A than that observed in SAGFF70A (Fig. 3b, c).

**Fig. 6:**
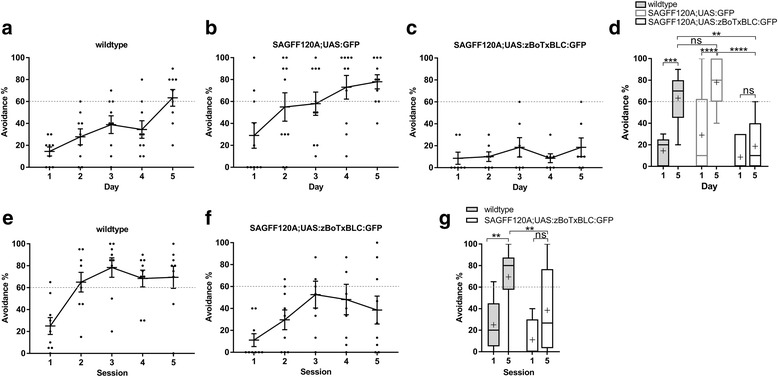
**a**–**d** Performance of two-way active avoidance responses of wild type, SAGFF(LF)120A;UAS:GFP, and the SAGFF120A;UAS:zBoTxBLC:GFP fish in blind experiments. Wild type fish (*n* = 9) (**a**), SAGFF(LF)120A;UAS:GFP fish (*n* = 10) (**b**), and SAGFF120A;UAS:zBoTxBLC:GFP fish (*n* = 7) (**c**) were analyzed for active avoidance fear conditioning under blind conditions, in which the fish identities were not known to the experimenter. Sibling fish were used for SAGFF120A;UAS:GFP and SAGFF120A;UAS:zBoTxBLC:GFP. Means ± SEM and avoidance (%) for individual fish are plotted. **d** Comparison of performance of avoidance responses at days 1 and 5 in Tukey box plot. Mean is marked by ‘+’. Two-way ANOVA, genotype (wild type, double transgenic) × trial number (day 1, day 5) (*F* = 9.082, *P* = 0.0005), and Tukey’s multiple comparison post-hoc tests were performed (**P < 0.01, ***P < 0.001, ****P < 0.001; ns, not significant). Both wild type and SAGFF(LF)120A;UAS:GFP fish exhibited active avoidance. SAGFF120A;UAS:zBoTxBLC:GFP fish showed a significantly reduced performance. **e**–**f** Performance of the active avoidance response in 1-day conditioning. One session was composed of 20 trials and five sessions were conducted within 1 day. Wild type fish (*n* = 9) could perform avoidance responses and SAGFF120A;UAS:zBoTxBLCGFP fish (*n* = 9) showed reduced performance. Means ± SEM and avoidance (%) for individual fish are plotted. Two-way ANOVA, genotype (wild type, double transgenic) × trial number (S1, S5) (*F* = 12.05, *P* = 0.0015), and Tukey’s multiple comparison post-hoc tests were performed (**P < 0.01)

To further confirm the behavioral phenotype observed in SAGFF120A;UAS:zBoTxBLC:GFP transgenic fish, we performed blind experiments for active avoidance fear conditioning using wild type, SAGFF120A;UAS:GFP, and SAGFF120A;UAS:zBoTxBLC:GFP fish (Fig. 6), in which the fish identities were not known to the experimenter. In these experiments, sibling fish were used for SAGFF120A;UAS:GFP and SAGFF120A;UAS:zBoTxBLC:GFP fish. While wild type and SAGFF120A;UAS:GFP fish could perform the conditioned active avoidance efficiently (Fig. 6a, b), SAGFF120A;UAS:zBoTxBLC:GFP fish exhibited significantly reduced performance in the avoidance response (Fig. 6c, d). Additionally, this result excluded the possibility that the transposon insertion in the SAGFF120A transgenic fish itself, but not the neurotoxin gene expression induced by Gal4FF, was the cause for the behavioral deficits.

To examine whether SAGFF120A;UAS:zBoTxBLC:GFP fish had a deficit in acquisition of active avoidance conditioning, but not in possible consolidation processes during the night, we developed another experimental procedure, in which five sessions (20 trials for active avoidance conditioning per session) were conducted within 1 day. Additionally, with this procedure, wild type fish could perform the avoidance response in more than 60% of the trials after the second session, while SAGFF120A;UAS:zBoTxBLC:GFP fish exhibited a much reduced performance (Fig. 6e–g), indicating that double transgenic fish had a deficit prominently in the acquisition process.

### Other behavioral phenotypes in SAGFF120A;UAS:zBoTxBLC:GFP fish

**Fig. 7:**
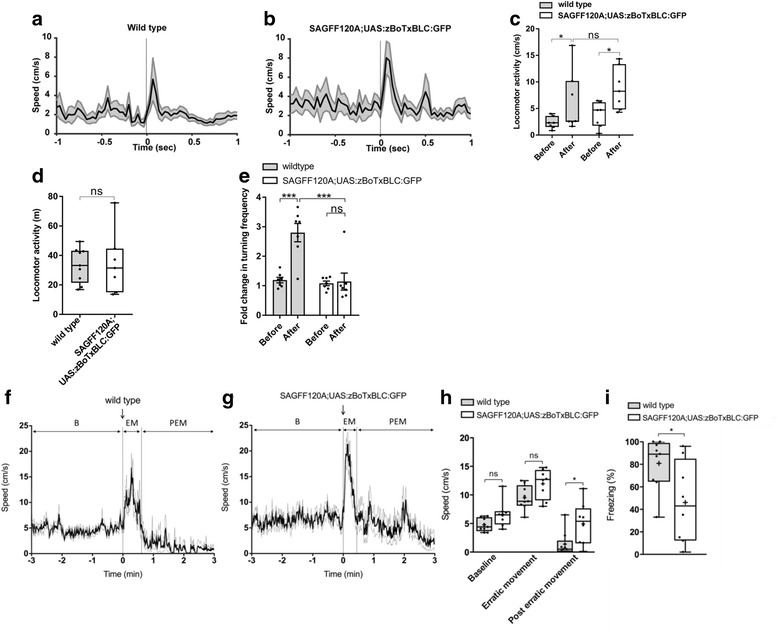
Behavioral analyses of the SAGFF120A;UAS:zBoTxBLC:GFP fish. **a**–**c** The light response of wild type (*n* = 7) (**a**) and the SAGFF120A;UAS:zBoTxBLC:GFP (*n* = 7) (**b**) fish. Means ± SEM are plotted. **c** The maximum locomotor activities 100 ms before (Before) and after (After) light-on are plotted with Tukey box plot. Wilcoxon signed rank test was performed for the same group (*P* = 0.0313 for wild type and *P* = 0.0156 for SAGFF120A;UAS:zBoTxBLC:GFP fish). Mann–Whitney U test was performed between two groups (*P* = 0.2086 for “Before”, *P* = 0.2593 for “After”). ns, not significant. **d** Comparison of the locomotor activity of wild type (*n* = 9) and SAGFF120A;UAS:zBoTxBLC:GFP (*n* = 7) fish with Tukey box plot. The Mann–Whitney U test was performed (*P* = 0.8372). **e** Pavlovian fear conditioning. Wild type fish (*n* = 7) showed increased turning activities in response to CS after conditioning. SAGFF120A;UAS:zBoTxBLC:GFP (*n* = 7) fish showed significantly reduced turning activities. Mean ± SEM and individual values are plotted. Two-way ANOVA, genotype (wild type, double transgenic) × conditioning (before, after) (*F* = 16.10, *P* = 0.0005), and Tukey’s multiple comparison post-hoc tests were performed (***P < 0.001). **f**–**i** The alarm response of wild type (*n* = 9) (**f**) and SAGFF120A;UAS:zBoTxBLC:GFP fish (*n* = 8) (**g**). (**f**, **g**) The speed of fish before and after addition of the skin extract. Mean ± SEM are plotted. The locomotor activities were divided into three phases; B (before addition of the skin extract), EM (erratic movement), and PEM (post-erratic movement). **h** Comparison of the speeds during B, EM, and PEM with Tukey box plot. Mean is marked by ‘+’. Unpaired *t*-test with Welch’s correction was performed (*P < 0.05). **i** Comparison of freezing during PEM. Mean is marked by ‘+’. Unpaired *t*-test with Welch’s correction was performed (*P < 0.05.)

To exclude the possibility that SAGFF120A;UAS:zBoTxBLC:GFP fish might have a deficit in the visual system, we analyzed the response to a light stimulus. Wild type fish showed increased swimming speed immediately after the CS (green LED) was turned on (within 100 ms) (Fig. 7a). A similar light response was observed in SAGFF120A;UAS:zBoTxBLC:GFP fish (Fig. 7b, c), indicating that the double transgenic fish could detect the green LED.

To exclude the possibility that SAGFF120A;UAS:zBoTxBLC:GFP fish might have a deficit in the motor system, we analyzed the locomotor activity during free swimming of wild type and double transgenic fish. We detected comparable levels of locomotor activity in both wild type and double transgenic fish (Fig. 7d).

The mammalian amygdala mediates both Pavlovian and active avoidance fear conditioning [1, 2]. Pavlovian fear conditioning has also been described in fish [10, 11]. We tested whether SAGFF120A;UAS:zBoTxBLC:GFP fish also show a deficit in Pavlovian fear conditioning. In this procedure, we placed fish in a small rectangular tank and gave US (electric shock) 9 s after CS (light) was on. After repeating US coupling with CS five times, CS was administered and the turn activity was measured. We found that, while wild type fish showed an approximately three-fold increase in the turn activity after training, SAGFF120A fish exhibited significantly reduced turn activities (Fig. 7e and Additional file 5: Movie S2), indicating that the Gal4FF-expressing cells in SAGFF120A were essential for both active avoidance and Pavlovian fear conditioning.


Additional file 5:**Movie S2.** Pavlovian fear conditioning of wild type fish. The movie shows an example of the analysis. Before conditioning: Wild type fish were placed in a white acrylic box and only CS (green LED) was given for 10 s. The turning activity during CS was measured. During conditioning: US (electric shock) was given for 1 s, 9 s after CS was on. After conditioning: CS was given for 10 s, and the turning activity was measured. (MOV 2802 kb)


In mammals, it has been reported that lesions in the basolateral amygdala lead to a deficit in an innate unconditioned response (freezing) to a natural dangerous stimulus (e.g., a ball of cat hairs for rats) [28]. It has been known that zebrafish display robust innate fear response to the alarm substance included in the skin extract, with fish exhibiting erratic movement followed by freezing [29, 30]. To test whether SAGFF120A;UAS:zBoTxBLC:GFP fish exhibit the alarm response, we analyzed locomotor activities of wild type (*n* = 9) and SAGFF120A;UAS:zBoTxBLC:GFP (*n* = 8) fish upon administration of the skin extract (Fig. 7f–i). Both wild type and double transgenic fish responded to the skin extract, and exhibited erratic movement (1.8- to 2.0-fold increase of the average speed) followed by freezing (Additional file 6: Movie S3). However, we detected significant differences in the average speed and freezing duration in the phase of post-erratic movement between wild type and SAGFF120A;UAS:zBoTxBLC:GFP fish; namely, SAGFF120A;UAS:zBoTxBLC:GFP fish showed an increased average speed and decreased freezing duration, suggesting that Gal4FF-expressing cells may play a role in modulation of the freezing behavior (Fig. 7h, i).


Additional file 6:**Movie S3.** Alarm responses of wild type fish and SAGFF120A;UAS:zBoTxBLCGFP fish. Behaviors of wild type and SAGFF120A;UAS:zBoTxBLCGFP fish were videotaped upon addition of skin extract. (MOV 8664 kb)


### Characterization of the neuronal population in Dm (120A-Dm neurons) essential for fear conditioning

**Fig. 8:**
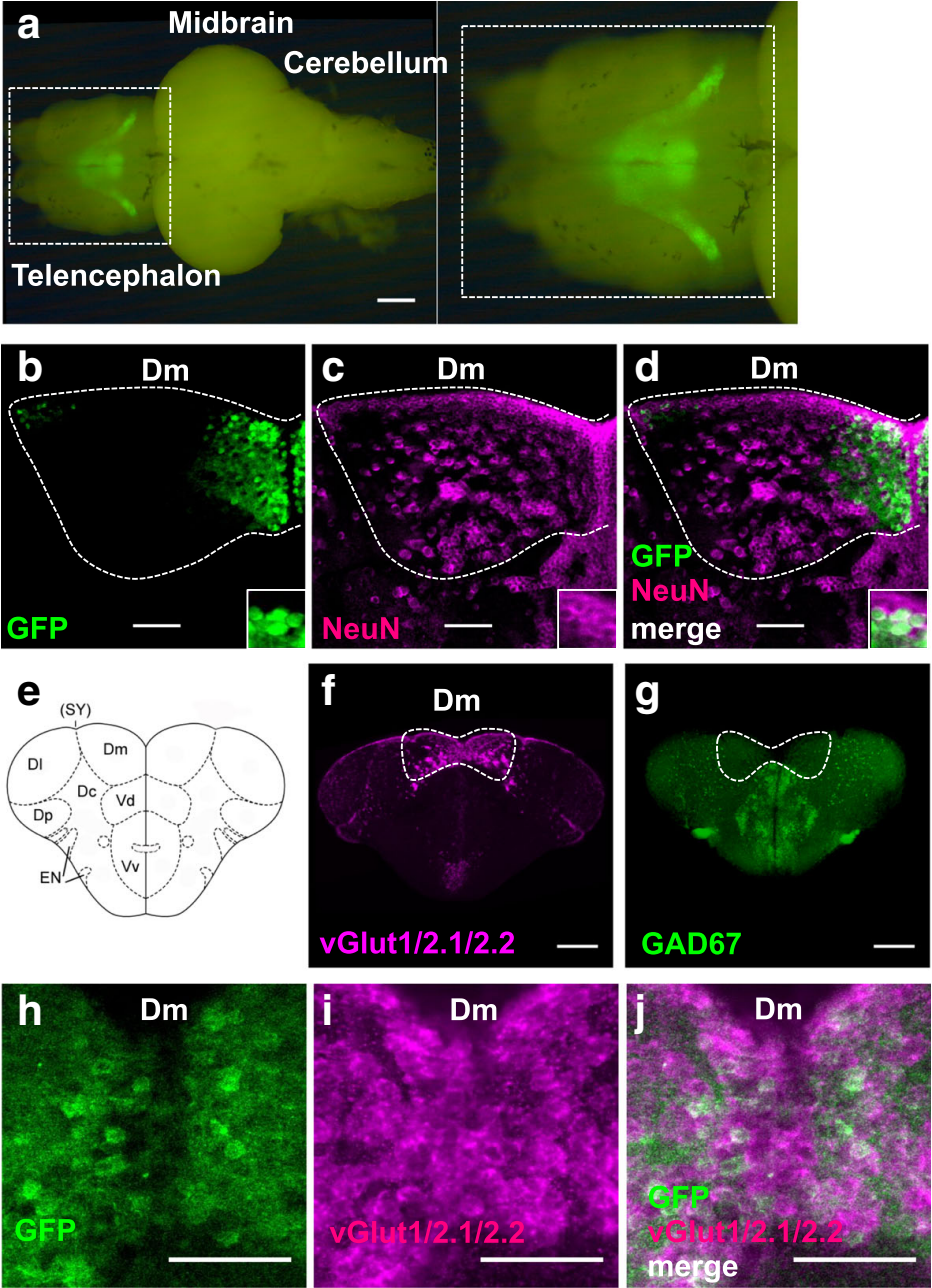
Immunohistochemical analyses of the 120A-Dm neurons. **a** Fluorescence imaging of the brain of SAGFF120A;UAS:GFP fish from the dorsal side. Dm, medial zone of the dorsal telencephalic area. Scale bars, 500 μm. **b**–**d** Double immunofluorescence staining of the telencephalon of SAGFF120A;UAS:GFP fish using the anti-GFP (green) (**b**) and anti-NeuN (magenta; neuronal marker) (**c**) antibodies. **d** A merged image; 16% (1986/12282) of NeuN-positive cells were GFP-positive and 99% (2394/2423) of GFP-positive cells were NeuN-positive. **e** A coronal view of the zebrafish telencephalon. Dm, medial zone of dorsal telencephalic area (D); Dl, lateral zone of D; Dc, central zone of D; Dp, posterior zone of D; SY, sulcus ypsiloniformis; Vd, dorsal nucleus of ventral telencephalic area (V); Vv, ventral nucleus of V; EN, entopeduncular nucleus. **f** Fluorescence in situ hybridization using the *vglut1/2.1/2.2* probes. **g** Fluorescence in situ hybridization using the *GAD67* probe. **h**–**j** Immunofluorescence staining of the telencephalon of SAGFF120A;UAS:GFP fish using anti-GFP (green) (**h**) and fluorescence in situ hybridization using *vglut1*/*2.1*/*2.2* probes (magenta) (**i**). **j** A merged image; 94% (352/374) of GFP-positive cells were glutamatergic. Scale bars, 200 μm (**f**, **g**) and 50 μm (**b**–**d**, **h**–**j**)

The GFP expression pattern in the SAGFF120A;UAS:GFP line showed characteristic features from a dorsal view; one group of GFP-positive cells was located in the ventromedial region, and the other was located in the dorsal region and extended laterally towards the posterior telencephalon (Fig. 8a).

To examine if GFP-positive cells are indeed neurons, we analyzed brain sections from SAGFF120A;UAS:GFP fish by immunohistochemistry using anti-GFP and anti-NeuN (neuronal marker) antibodies. Overall, 99% of GFP-positive cells were NeuN-positive (2394/2423), revealing that most of the GFP-positive cells were neurons (Fig. 8bd–d). We also found that 16% (1986/12282) of the NeuN-positive cells in the Dm area were GFP-positive, indicating that only a subpopulation of neurons in Dm were labeled in the SAGFF120A line. The GFP-positive (Gal4FF-positive) cells in the Dm area in the SAGFF120A line are hereafter referred to as 120A-Dm neurons.

We then performed in situ hybridization using the *vglut1*/*2.1*/*2.2* and *gad67* probes. In the zebrafish telencephalon, the pallium (dorsal telencephalon) and subpallium (ventral telencephalon) were rich in glutamatergic and GABAergic neurons, respectively (Fig. 8e–g), in agreement with previous reports [14, 15]. We analyzed brain sections from SAGFF120A;UAS:GFP fish using the anti-GFP antibody and *vglut1*/*2.1*/*2.2* probes, and found 94% (352/374) of the GFP-positive cells to be glutamatergic (Fig. 8h–j).

### Characterization of projections of the 120A-Dm neurons

**Fig. 9:**
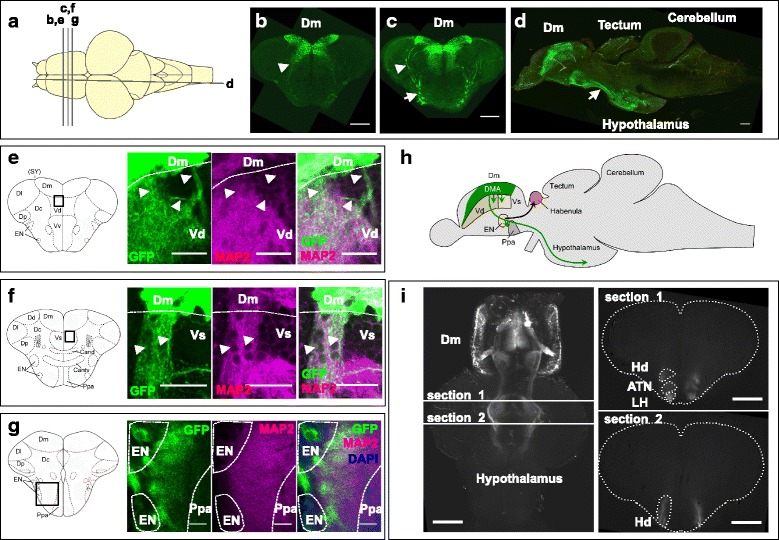
Projections of 120A-Dm neurons. **a** Dorsal view of the brain. The positions of the sections are shown as bars (**b**–**g**). **b**–**d** Immunofluorescence staining using anti-GFP. **b**, **c** Coronal sections of SAGFF70A;UAS:GFP;UAS:zBoTxBLC:GFP fish. **d** Sagittal section of SAGFF120A;UAS:GFP;UAS:zBoTxBLC:GFP fish. Axons of the 120A-Dm neurons project to the hypothalamus area. Arrowheads in **b** and **c** indicate projections from two groups of the 120A-Dm neurons. Arrows in **c** and **d** indicate the lateral forebrain bundle. **e**–**g** Double immunofluorescence staining using anti-GFP (green) and anti-MAP2 (magenta; dendritic marker) of the brain of SAGFF120A;UAS:GFP fish. Schemes of coronal views of the telencephalon are shown on the left. **e** Projections to Vd. **f** Projections to Vs. **e**, **f** Arrowheads mark projections that were not co-stained with anti-MAP2. **g** Projections to the neuropil area of EN and Ppa. Cell bodies in EN and Ppa were stained with DAPI (blue). Dm, medial zone of dorsal telencephalic area (D); Dl, lateral zone of D; Dc, central zone of D; Dp, posterior zone of D; SY, sulcus ypsiloniformis; Vd, dorsal nucleus of ventral telencephalic area (V); Vv, ventral nucleus of V; EN, entopeduncular nucleus; Ppa, Parvocellular preoptic area; Cand, Commissura anterior, pars dorsalis; Cantv, Commissura anterior, pars ventralis. **h** A schematic view of axonal projections of the 120A-Dm neurons (green) to Vd, Vs, EN, Ppa, and the hypothalamus. **i** Light-sheet microscopy of a cleared brain from the SAGFF120A;UAS:GFP fish. Horizontal section (left) and coronal sections (right) showed projections of the 120A-Dm neurons terminated in the lateral hypothalamic nucleus (LH), the anterior tuberal nucleus (ATN), and dorsal zone of periventricular hypothalamus (Hd). Scale bars, 200 μm (**b**–**d**), 50 μm (**e**, **g**), 25 μm (**f**), and 500 μm (**i**)

To investigate projections of the 120A-Dm neurons, we first created serial cross sections and sagittal sections of the brain from SAGFF120A;UAS:GFP fish, and analyzed them by immunohistochemistry using the anti-GFP antibody and confocal microscopy (Fig. 9a–d). We found that the major projections from the 120A-Dm neurons proceeded ventrally within the telencephalon, joined the lateral forebrain bundle, proceeded more posteriorly, and terminated in the hypothalamic area (Fig. 9d). We also observed projections of the 120A-Dm neurons toward the subpallium area, including the dorsal nucleus (Vd) and the supracommissural nucleus of the ventral telencephalic area (Vs). Some of these projections were not co-immunostained with the anti-MAP2 antibody (dendritic marker) (Fig. 9e, f) [31], suggesting that they were axonal projections. In addition, we observed neurites of the 120A-Dm neurons spread in the neuropil areas of the entopeduncular nucleus (EN) and the preoptic area, suggesting connections to these nuclei (Fig. 9g, h).

Further, we prepared a cleared brain sample from SAGFF120A;UAS:GFP fish by the Scale method [32], and analyzed it with light-sheet microscopy. This analysis also visualized the projections from the Dm area to the ventral telencephalon and hypothalamus. In the hypothalamic area, the projections proceeded laterally and terminated in the lateral hypothalamic nucleus (LH), the anterior tuberal nucleus (ATN), and dorsal zone of periventricular hypothalamus (Hd) (Fig. 9i and Additional file 7: Movie S4).


Additional file 7:**Movie S4.** 3D image of the brain from SAGFF120A;UAS:GFP fish. A GFP fluorescence image of a transparent brain from SAGFF120A;UAS:GFP fish analyzed by light-sheet microscopy is shown. (MOV 6882 kb)


**Fig. 10:**
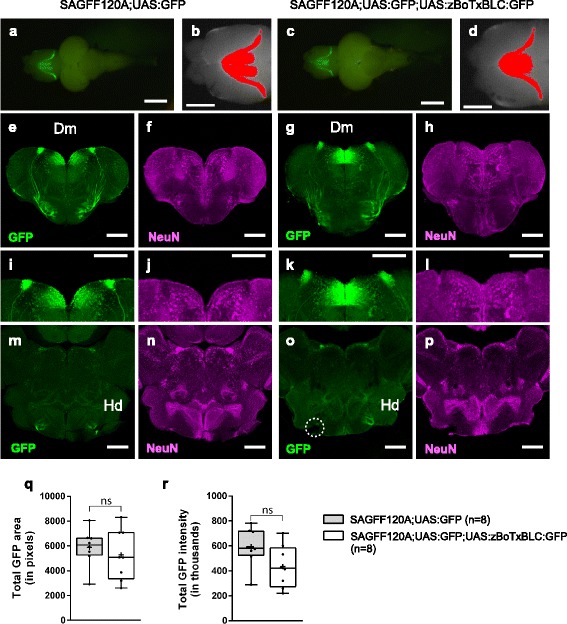
GFP expression patterns in SAGFF120A;UAS:GFP and SAGFF120A;UAS:GFP;UAS:zBoTxBLC:GFP fish. **a**–**d** GFP fluorescence in the dorsal view of the brains from SAGFF120A;UAS:GFP (~10 months old; #4 in Additional file 8: Figure S4) (**a**, **b**) and SAGFF120A;UAS:GFP;UAS:zBoTxBLC:GFP (~10 months old; #4 in Additional file 8: Figure S4) fish (**c**, **d**). Areas having more GFP intensity than background (the maximum intensity measured in the posterior part of the telencephalon) were identified by using ImageJ [57] and shown in red (**b**, **d**). **c**–**p** Immunohistochemistry using anti-GFP (green; **e**, **g**, **i**, **k**, **m**, **o**) and anti-NeuN (a neuronal marker, magenta; **f**, **h**, **j**, **l**, **n**, **p**). Coronal sections of the telencephalon (**e**–**l**) and the hypothalamus (**m**–**p**) of SAGFF120A;UAS:GFP (**e**, **f**, **i**, **j**, **m**, **n**) and SAGFF120A;UAS:GFP;UAS:zBoTxBLC:GFP (**g**, **h**, **k**, **l**, **o**, **p**) fish. **i**–**l** Magnified images of **e**–**h**. GFP-positive cells in Dm, and projections from these cells to the target area in Hd (dorsal zone of periventricular hypothalamus) were detected. A dotted circle in **o** indicated a broken part. Scale bars: 1 mm (**a**, **c**), 500 μm (**b**, **d**), and 200 μm (**e**–**p**). **q**–**r** Comparisons of total GFP intensity (**q**) and area (**r**) data obtained by ImageJ analysis of SAGFF120A;UAS:GFP (*n* = 8) and SAGFF120A;UAS:GFP;UAS:zBoTxBLC:GFP (*n* = 8) fish (Additional file 8: Figure S4) are plotted with Tukey box plot. Unpaired *t*-test with Welch’s correction was performed between these transgenic fish (ns, not significant)

It should be noted that, in SAGFF120A;UAS:zBoTxBLC:GFP fish, the neurotoxin gene could be expressed from the larval to adult stages. To examine if any gross morphological differences were generated due to the possible continuous neurotoxin expression, we analyzed the brains from SAGFF120A;UAS:GFP and SAGFF120A;UAS:GFP;UAS:zBoTxBLC:GFP fish (Fig. 10, Additional file 8: Figure S4). First, we observed similar shapes of GFP expression patterns in the dorsal view of the telencephalon (Fig. 10a, c), and analyzed their intensities and the areas (Fig. 10b, d) and could not detect significant differences in the total GFP intensity and areas between these transgenic lines (*n* = 8 for each transgenic lines; Fig. 10q, r). We then analyzed coronal sections from these brain samples by immunohistochemistry using GFP and NeuN antibodies, and could detect GFP-positive cells in the Dm area (Fig. 10e–l) and GFP-positive projections toward the ventral telencephalon from these cells that reached the target area in the Hd (dorsal zone of periventricular hypothalamus) in both of these transgenic lines (Fig. 10m–p). Further, the NeuN staining of these areas also showed similar patterns (Fig. 10f–p). Thus, we could not detect gross morphological differences in the GFP-positive and surrounding areas in SAGFF120A;UAS:GFP and SAGFF120A;UAS:GFP;UAS:zBoTxBLC:GFP fish at these levels of analysis.

## Discussion

### Functional and neurochemical similarities between the 120A-Dm neurons and the pallial amygdala nuclei

It has been postulated that the medial zone of the dorsal telencephalon (Dm) in fish is a homolog of the mammalian amygdala based on neuroanatomical studies [12, 16, 17] and ablation experiments in goldfish [18]. However, the neuronal population and circuitry had not yet been identified. In the present study, we performed a genetic approach using zebrafish and, for the first time, identified a subpopulation of neurons located in the Dm area, which we named 120A-Dm neurons, essential for acquisition of both active avoidance and Pavlovian fear conditioning.

The mammalian amygdala consists of pallial (cortical) and subpallial (striatal) portions, and is further subdivided into multiple nuclei. The BLA, which are included in the pallial portion, contain predominantly glutamatergic neurons and are essential for the CS–US association [3]; namely, it was shown that the BLA lesions caused deficits in both Pavlovian and active avoidance fear conditioning [1, 5, 33]. The 120A-Dm neurons identified in the teleost Dm were also mostly glutamatergic and essential for both Pavlovian and active avoidance fear conditioning. From these functional and neurochemical similarities, we suggest that the 120A-Dm neurons are the functional equivalent of the pallial amygdala, and presumably neurons in BLA. It is not known whether the entire population of the 120A-Dm neurons or only part of them are essential for fear conditioning. Further subdivision of the 120A-Dm neurons will be required to answer this question.

In mammals, BLA lesions cause a deficit in an innate unconditioned response to a natural dangerous stimulus (for instance, cat hairs for rats) [28]. Additionally, there was a contradictory report describing that inactivation of BLA impaired the learned, but not innate, fear response in rats [34]. Herein, we tested whether the 120A-Dm neurons are involved in the innate fear response in zebrafish by analyzing reactions to a skin extract [29, 30]. Similarly to wild type fish, SAGFF120A;UAS:zBoTxBLC:GFP fish could respond to the skin extract and perform erratic movement and freezing. However, during the post-erratic movement phase, fish showed reduced freezing behaviors in comparison to wild type fish, suggesting that 120A-Dm neurons may play a role in modulation of the freezing behavior. Consistent with this, it was reported that cells in the Dm are activated upon administration of a skin extract [35]. Further, it has been shown that the alarm response- or alarm substance-induced fear conditioning can be modulated by social buffering [36] or administration of the endocannabinoid receptor CB1 agonist [35]. Transgenic fish that expressed Gal4FF in 120A-Dm neurons should allow us to investigate a neuronal basis of these behaviors as well as other motivational and emotional behaviors in zebrafish, such as light/dark choice or drug-seeking behaviors [19, 20], that are thought to be mediated by the amygdala-like functions of Dm.

In addition to the Dm, the present study highlighted other forebrain regions possibly important for fear conditioning. For instance, we found a relatively large number of lines that showed reduced avoidance responses and that commonly had Gal4FF expression in the ventral nucleus of the ventral telencephalon (Vv) or the preoptic area. Vv has been postulated to be a homolog of the septal nuclei of mammals [13, 37], which also play a crucial role in fear learning [38]. The preoptic area has been postulated to be a homolog of the mammalian paraventricular nucleus of the hypothalamus (PVN) [39], containing the magnocellular neurosecretory system that mediates fear responses [40]. hspGFFDMC56B fish had rather specific Gal4FF expression in the preoptic area and should be used for further studies to explore their role in fear conditioning.

### *emx3*-expressing neurons are essential for fear conditioning

In the SAGFF70A and SAGFF120A lines, Gal4FF was expressed in a pattern similar to that of the *emx3* gene. The zebrafish *emx3* gene is expressed in the dorsal telencephalon at the embryonic stage and in the dorsomedial region in the adult brain [25–27]. Knockdown of the *emx3* function by morpholino impairs expression of dorsal telencephalic marker genes [41]. However, the function of the *emx3*-expressing cells had not been analyzed. The present study revealed the role of the *emx3*-expressing neurons in fear conditioning. It should be noted that, in our approach, the botulinum neurotoxin was continuously expressed throughout development. Although we could not detect gross morphological changes in the GFP-positive neurons in SAGFF120A;UAS:GFP;UAS:zBoTxBLC:GFP fish, a conditional system that prohibits neuronal activities only in the adulthood is required to examine the possibility that the toxin expression during developmental stages may have caused the observed behavioral deficits. Efforts are currently in progress along this line.

The mouse genome has two paralogs, *Emx1* and *Emx2* [42]. *Emx1* is expressed in the dorsal telencephalon in the developing brain, and *Emx1*-expressing cells give rise to excitatory neurons in the pallium, including pallial portions of the amygdala [43]. *Emx2* is expressed earlier and more broadly, and plays a major role in the formation of the medial limbic cortex [44]. The *Emx1-*expressing cells in the developing telencephalon in chicken and *Xenopus* also contribute to cells in amygdalar nuclei [45, 46]. Thus, roles of cells expressing the *emx* family genes in the developing brain may be conserved during vertebrate evolution. However, the function of *Emx*-expressing cells in the adult brain was not characterized. It should be interesting to investigate *Emx*-expressing neurons in the adult brain in other vertebrates to see whether those neuronal populations harbor essential roles in fear conditioning as well.

### Projections of 120A-Dm neurons to the hypothalamus

We found that 120A-Dm neurons had major projections to the hypothalamic area. In mammals, the hypothalamus is important in fear responses, controlling heart rate and blood pressure [1, 2]. We assume that the Dm–hypothalamus connection should also play an important role in mediating fear responses in fish. In previous work using goldfish, efferent projections from Dm to the hypothalamic area, including the ATN and dorsal zone of periventricular Hd, were identified by anterograde labeling [17], and minor outputs from Dm to the LH were detected by retrograde labeling [47]. The present study clearly visualized projections of 120A-Dm neurons that terminated in the hypothalamic area, including ATN, LH, and Hd, consistently with the results obtained in the tracer experiments in goldfish.

In mammals, the CeA is the major output center, has projections to the lateral hypothalamus, and predominantly contains GABAergic neurons [1, 2, 5]. In contrast, 120A-Dm neurons are mostly glutamatergic and thought to be a functional equivalent of the pallial amygdala. Further studies on the roles of excitatory projections from Dm to the hypothalamus should provide new insights into the conservation and diversification of limbico-hypothalamic connections during evolution.

### Projections of 120A-Dm neurons to the other telencephalic regions

We found the projection of 120A-Dm neurons also terminated in the neuropil area of the EN and preoptic area, Vd, and Vs, suggesting that these are possible targets. The EN consists of a dorsal GABAergic part and a ventral glutamatergic part, which have been hypothesized to be homologous to the EN of non-primate mammals (internal segment of the globus pallidus of primates) and the bed nucleus of the stria medullaris, respectively [14]. In goldfish and zebrafish, the ventral glutamatergic neurons have been shown to project to the Hb nuclei [11, 48], and it was recently shown that the Hb-median raphe circuit in zebrafish is essential for active avoidance conditioning [11]. Thus, we hypothesize that the projection of 120A-Dm neurons to the EN may play a role in mediating active avoidance responses. Consistent with this hypothesis, hspGFF38B and hspGFF55B fish, which strongly expressed Gal4FF in the Hb and EN, respectively, also exhibited deficits in active avoidance conditioning when crossed with the UAS:neurotoxin line (Additional file 2: Figure S1). Further analyses using these transgenic fish should reveal the role of the Dm-EN-Hb circuit in active avoidance responses. Vd and Vs are structures located in the subpallium and rich in GABAergic neurons, and have been hypothesized as homologs of the mammalian striatum and CeA, respectively [14]. Thus, these connections may correspond to connections of BLA to the striatum, and the intra-amygdaloid connection of BLA to CeA, which have been described in mammals [1, 2, 49].

### The genetic approach reveals functional neuronal circuits mediating adult behavioral phenotypes

Herein, we succeeded in performing a genetic approach to study a learning behavior in zebrafish. We think this success mainly relies on three factors. Firstly, our trap lines expressed Gal4FF specifically and strongly in the adult brain. We have shown that Gal4FF is a strong but less-toxic transcription activator in zebrafish [21, 50] and, since then, performed gene and enhancer trap genetic screens to label specific cell types by Gal4FF expression at the larval stages [51]. The present study demonstrated that the gene and enhancer trap approaches are applicable to generate specific Gal4FF expression patterns at the adult stage as well. Secondly, the UAS:zBoTxBLC:GFP line expressed the neurotoxin reliably and reproducibly in combination with various Gal4FF lines. It was reported that some UAS effector lines, especially those containing 14xUAS, suffered from silencing effects [52]. In contrast, we have been using 5xUAS for UAS-effector lines [21], and have not experienced such severe silencing effects. Further, when we created UAS:zBoTxBLC:GFP fish, we generated more than 30 different insertions, selected transgenic fish that showed the strongest expression by crossing them with several different Gal4FF driver lines, and established the best line with a single transposon insertion. The UAS:zBoTXBLC:GFP line thus established worked effectively in the larval stages [24] as well as in the adult stage (this study). Thirdly, our protocol developed for active avoidance fear conditioning has worked very efficiently and reproducibly, enabling the identification of transgenic lines with reduced learning activities out of many candidate lines. In summary, the present study demonstrated that the genetic approach combined with a behavioral paradigm is powerful to dissect functional neuronal circuits in the adult zebrafish brain, and should be applicable to the study of other brain circuits and behaviors.

## Conclusions

Fear conditioning is commonly observed in vertebrate species. In teleost, it has been postulated that the medial zone of the dorsal telencephalon (Dm) is a homolog of the mammalian amygdala, and essential for retention of the conditioned avoidance responses. However, Dm is a broad area and functional neuronal populations had not yet been identified. Herein, we identified a subpopulation of neurons in Dm essential for fear conditioning through a genetic approach in zebrafish. These neurons are mostly glutamatergic and have projections to other brain regions, including the hypothalamic area and ventral telencephalon. We propose that these should be functional equivalents of neurons in the mammalian pallial amygdala, mediating a CS–US association. Thus, we established a basis for understanding the evolutionary conservation and diversification of functional neural circuits mediating fear conditioning in vertebrates.

## Methods

### Fish

Transgenic fish that expressed Gal4FF were generated by the gene trap and enhancer trap methods [21]. Transposon integration sites in transgenic fish lines were analyzed by Southern blot hybridization and inverse PCR as described previously [53]. UAS:GFP fish were used to visualize Gal4FF expression [21]. The T2SUASzBoTXBLCGFP construct containing 5xUAS, TATA sequence, the codon-optimized botulinum toxin B light chain gene [54] fused to the *EGFP* gene, and SV40 polyA between *cis*-sequences of *Tol2* was created and injected to fertilized eggs to generate the UAS:zBoTXBLC:GFP transgenic fish.

### Analysis of Gal4FF expression in the adult brain

The GFP expression patterns in the adult brain were first observed under a fluorescence microscope (MZ 16FA, Leica Microsystems). The heads were then fixed in 4% paraformaldehyde and dissected to take the brains out of the skulls as described previously [55]. The isolated brains were observed under a fluorescence microscope (MZ 16FA, Leica Microsystems). The fixed brain samples were embedded in 1% agarose in 0.1 M phosphate buffered saline (PBS; pH 7.4) and 100 μm-thick serial coronal sections were made by using a vibratome. The slices were collected in 24-well plates and mounted on slide glasses (Matsunami) using PermaFluor Aqueous Mounting Medium (Thermoscientific). Sections were observed under an upright epifluorescence microscope (Axio Imager Z1, Zeiss).

### Preparation of fish for behavioral analyses

Fish aged from 5 months to 1.5 years were used for behavioral studies. Prior to behavioral assays, fish were moved to a behavioral assay room and kept in isolation in 2-L tanks for 2 days.

### Two-way active avoidance fear conditioning (5-day procedure)

A white opaque acrylic tank (length 41 cm × width 17 cm × height 12 cm) with transparent walls at both ends, a trapezoidal wedge (10 cm at the top and 20 cm at the bottom × width 17 cm × height 5 cm) in the center of the tank, green LEDs (3.3 V DC, 2 A), and a pair of platinum mesh electrodes (12 V AC) were used. Behaviors were monitored and analyzed by programs created by using LabView 8.6 (National Instruments). Habituation was performed for 15 min per day for 2 days in the shuttle box. Conditioning involved (1) 2 min in the shuttle box; (2) initiating CS (green LED) and US 10 s later (12 V AC electric shock); and (3) turning off of CS and US 5 s after initial US; (4) if fish escaped while CS was on, US was not given; and (5) if the fish moved to another compartment while US was on, both CS and US were turned off. The process was repeated 10 times with an inter-trial interval of 25 ± 5 s per day on 5 consecutive days. The avoidance index was calculated as the number of successful escape responses per 10 trials. The two-way active avoidance conditioning was also performed as blind experiments, in which the fish identities were not known to the experimenter (Fig. 6).

### Two-way active avoidance fear conditioning (1-day procedure)

The same setup as the 5-day procedure was used. Habituation was performed for 1 h per day for 2 days in the shuttle box. Conditioning involved (1) 5 min in the shuttle box; (2) initiating CS and US 10 s later (9 V AC electric shock); (3) turning off of both CS and US 5 s after US; (4) if fish escaped while CS was on, US was not given; and (5) if the fish moved to another compartment while US was on, both CS and US were turned off. The process was repeated 20 times with intervals of 25 ± 5 s per session, and five sessions were performed with an inter-trial interval of 3 min. The avoidance index was calculated as the number of successful escape responses per 20 trials.

### The light response

The response to a light stimulus, which was described previously for larval zebrafish [56], was measured by using adult fish. A white opaque acrylic box (length 12 cm × width 17 cm × height 12 cm) with a transparent wall on one-side and equipped with a green LED light (3.3 V, 2A DC) was used. The water level was 5 cm in depth. Fish behavior was monitored and analyzed by programs created using LabView 8.6 (National Instruments). Fish were kept in the apparatus for 10 min and the green LED light was then turned on for 10 s. Fish locomotion was recorded at 27 fps, and the locomotor activities (speed) of the fish 100 ms before and after light-on were analyzed. Movie analysis was carried out with ImageJ 1.48v (US National Institute of Health) [57].

### Locomotor activity

Fish were habituated for 5 min in an opaque tank (length 33 cm × width 19 cm × height 15 cm). Behaviors were recorded for 10 min and analyzed by using a program created with LabView 8.6 (National Instruments). Locomotor activities were calculated as distances travelled in 10 min.

### Pavlovian fear conditioning

A white opaque acrylic box (length 12 cm × width 17 cm × height 12 cm) with a transparent wall on one-side equipped with a green LED light (3.3 V, 2A DC) and a pair of platinum mesh electrodes (9 V AC) was used. Behaviors were monitored and analyzed by programs created with LabView 8.6 (National Instruments). Habituation was performed by (1) placing fish for 10 min in the tank, (2) then initiating CS for 10 s, five times, at 50-s intervals, (3) followed by 10 min of free-swimming. Conditioning involved initiating CS and, 9 s later, initiating US for 1 s, followed by turning off of both CS and US. The process was repeated 5 times at 50-s intervals and the entire conditioning process was repeated once. The test involved (1) 10 min of free-swimming after training and (2) delivery of CS five times at 50-s intervals. The behavior was recorded at 27 fps and the movies were analyzed with ImageJ [57]. Turning angles were determined by measuring the change of the head-tail axis of the fish. Turning with angles greater than 90° within six frames (0.22 s) was defined as a conditioned response. The number of conditioned responses during the 10 s before and after CS was counted, and fold changes in turning frequencies were calculated with or without conditioning.

### The alarm response

Skin extracts were prepared fresh and kept on ice on the day of use [30]. Adult fish (> 3 months old) were anesthetized in Tricaine (0.025%) and quickly sacrificed by decapitation. Excess water was removed from the skin using a paper towel, and 15 shallow cuts were made on each side of the trunk, avoiding contamination of the blood. Cuts were washed with distilled water, and 10 mL of skin extract were collected from each fish. To test alarm response, fish were habituated for 10 min in a 2-L tank (length 25 cm × width 6.5 cm × height 16 cm) equipped with delivery tubes at both ends. Then, 2 mL of the skin extract was applied to the tank. Behavior was recorded for 3 min before and after addition of the skin extract. The locomotor activity of wild type and the double transgenic fish was divided into three phases, namely baseline (B, before addition of the skin extract), erratic movement (from addition of the skin extract to the time when the average speed dropped to the baseline level; 38 s for wild type and 24 s for the double transgenic fish), post-erratic movement, and analyzed. Freezing was defined as locomotor activities with a speed of less than 5 mm/s. The movies were analyzed using ImageJ [57].

### Statistical analyses

For active avoidance performance (5-day procedure), analysis of variance (ANOVA) and Dunnet’s multiple comparison tests were performed to test the statistical significance between test samples and control. For active avoidance performance (1-day procedure), ANOVA and Tukey’s multiple comparison tests were performed to test the statistical significance between test samples and control. For locomotive activity, Kruskal–Wallis test was performed. In Pavlovian fear conditioning, two-way ANOVA and Tukey’s multiple comparison post-hoc tests were performed. No data points were excluded in these analyses. All the statistical tests were performed by using PRISM 6 (GraphPad software).

### Immunohistochemistry and in situ hybridization

Coronal/sagittal sections of 100 μm in thickness were used for immunohistochemistry. The samples were treated for 1 h at room temperature in blocking buffer, 0.5% Tween-20 or Triton X-100, 3% bovine serum albumin (Sigma) in PBS, and then incubated overnight with primary antibodies diluted in blocking buffer at 4 °C. The samples were then washed with 0.5% Tween-20 or Triton X-100 in PBS and incubated with secondary antibodies. Rabbit anti-GFP polyclonal (1:500 dilution, A6455; Invitrogen, RRID:AB_221570, LOT number = 1,650,113), mouse anti-NeuN monoclonal (1:200 dilution, MAB377; Millipore, RRID:AB_2298772, LOT number = 2,428,671), and mouse monoclonal anti-MAP2 antibody [AP-20] (1:300 dilution, ab11268; Abcam, RRID:AB_297886) were used for the primary antibodies. AlexaFluor 488 goat anti-rabbit IgG (1:400 dilution, A11008; Invitrogen, RRID:AB_143165) and AlexaFluor 633 goat anti-mouse IgG (1:1000 dilution, A21050; Invitrogen, RRID:AB_141431) were used for the secondary antibodies. For in situ hybridization, digoxigenin-labeled probes were synthesized by using the *emx3* and the *vglut1/2.1/2.2* cDNAs as templates. In situ hybridization was performed on coronal slices of fixed brain using a protocol described previously [58] with modifications. Prehybridization and hybridization were performed at 65 °C for 2 h in Hyb(+) solution or at 65 °C overnight in Hyb(+) solution containing approximately 100 ng of digoxigenin-labeled probes, respectively. The samples were washed with 66% Hyb (−)/2X SSCT at 65 °C for 30 min, 33% Hyb(−)/2X SSCT at 65 °C for 30 min, 2X SSCT at 65 °C for 15 min, and with 0.2X SSCT at 65 °C for 30 min twice. Hyb(−): 50% formamide, 5X SSC (Gibco), 0.1% Tween-20 (Pierce). Hyb(+): Hyb(−) with 5 mg/mL RNA purified from torula yeast (Sigma) and 50 μL/mL heparin (Sigma). The samples were then incubated in blocking solution (2% blocking reagent in PBST) (Roche) at 4 °C overnight and then incubated in 1:5000 dilution of anti-digoxigenin-AP Fab-fragments (11093274910; Roche, RRID:AB_514497) at 4 °C overnight. For *emx3,* signals were detected using BM purple (Roche). The reaction was stopped by washing with PBST. The slices were mounted on slide glasses (Matsunami) using glycerol-gelatin mounting medium and observed under a microscope (Imager Z1). Images were taken with an AxioCam MRc5 (Zeiss) camera and analyzed with Axio Vision Ver4.1 imaging software (Zeiss). For *vglut1/2.1/2.2*, signals were detected with Fast Red Tablets (Roche). The slices were mounted on slide glasses using PermaFluor Aqueous Mounting Medium (Thermoscientific) and observed with a laser scanning confocal microscope (FV-1000-D; Olympus) or a Zeiss confocal microscope with Yokogawa CSU-W1 laser scanning unit (Yokogawa). Images were processed with ImageJ [57].

### Preparation of a cleared brain and light-sheet microscopy

The Scale method [32] was applied in the preparation of a cleared brain from SAGFF120A;UAS:GFP fish. The head was fixed with 4% paraformaldehyde/PBS (Wako) at 4 °C for overnight, and then dissected. The brain sample was washed with PBS, incubated in 20% sucrose/PBS at 4 °C for 2 days, embedded in OCT compound (Sakura), and frozen with liquid nitrogen. The sample was then thawed and washed with PBS, transferred into the ScaleA2 solution, and kept at 4 °C for more than 3 weeks. The ScaleA2 solution was changed every 2 days. The sample was observed with a light sheet fluorescence microscope (Zeiss Light sheet Z.1) with a 5× NA 0.16 lens. Fluorescence was measured with excitation (488 nm) and emission (SP 550 (Ch1) and LP585 (Ch2)) filters, and the GFP signal was obtained by subtracting Ch2 from Ch1. For image analysis, ZEN (Zeiss) and IMARIS 7.0 (Bitplane) were used.

## Additional files

Additional file 2: **Figure S1.** Performance of two-way active avoidance response of double transgenic (Gal4FF;UAS:zBoTxBLC:GFP) fish. The following Gal4FF transgenic fish lines were crossed with UAS:zBoTxBLC:GFP effector fish, and analyzed for two-way active avoidance fear conditioning. **a** hspGGFF10C (*n* = 6), **b** hspGGFF20A (*n* = 10), **c** hspGFF38B (*n* = 10), **d** hspGFF55B (*n* = 6), **e** SAGFF81B (*n* = 12), **f** SAGFF226F(*n* = 9), **g** SAGFF233A (*n* = 9), **h** SAGFF234A (*n* = 11), **i** hspGFFDMC12A (*n* = 12), **j** hspGFFDMC56B (*n* = 10), **k** hspGGFF19B (*n* = 9), **l** hspGGFF19C (*n* = 9), **m** hspGFF62A (*n* = 5), **n** gata6SAGFF94A (*n* = 6), **o** SAGFF27C (*n* = 6), **p** SAGFF38A (*n* = 8), **q** SAGFF87C (*n* = 5), **r** SAGFF92A (*n* = 8), **s** SAGFF183A (*n* = 5), **t** SAGFF195A (*n* = 6), **u** SAGFF212C (*n* = 5), **v** SAIGFF170B (*n* = 10), **w** hspGFFDMC76A (*n* = 10), **x** hspGFFDMC85C (*n* = 11). Mean ± SEM and avoidance (%) for individual fish are plotted. Performance of wild type fish (*n* = 28) described in Fig. 2 is shown in dotted lines. Two-way ANOVA, fish groups (wild type fish treated by CS-US, wild type fish treated by CS only and double transgenic fish including fish described in Figs. 2 and 3 × training session days (day 1, day 5), was performed (*F* = 7.236, *P* < 0.0001). Dunnett’s multiple comparison post-hoc tests were performed between avoidance percentage of wild type fish and double transgenic fish on session day 1 and day 5. *P < 0.05, **P < 0.01, ***P < 0.001, ****P < 0.0001; ns, not significant (*P* > 0.05). a–j Reduced performance of the active avoidance response was observed. k–x Performance of the active avoidance response was not significantly different between wild type and the double transgenic fish. (PPTX 8767 kb)

Additional file 3: **Figure S2.** GFP expression patterns of 16 Gal4FF;UAS:GFP fish that showed reduced performance of the active avoidance response. A dorsal view, a ventral view, and a schematic side view with positions of coronal sections are shown on the top. Serial coronal sections with position numbers are shown in the bottom. **a** hspGGFF10C, **b** hspGGFF20A, **c** hspGFF38B, **d** hspGFF55B, **e** SAGFF36B, **f** SAGFF70A, **g** SAGFF81B, **h** SAGFF120A, **i** SAGFF226F, **j** SAGFF228A, **k** SAGFF231A, **l** SAGFF233A, **m** SAGFF234A, **n** SAGFF234D, **o** hspGFFDMC12A, **p** hspGFFDMC56B. Scale bars in whole brain images: 500 μm. Scale bars in coronal section images: 200 μm. (PDF 3264 kb)

Additional file 4: **Figure S3.** GFP expression patterns in SAGFF120A;UAS:GFP fish at embryonic stages. Bright field and fluorescent images of frontal and lateral views of SAGFF120A;UAS:GFP fish at 24, 48, 72, and 96 hpf. Scale bar, 200 mm. (PPTX 1417 kb)

Additional file 8: **Figure S4.** GFP expression patterns in SAGFF120A;UAS:GFP and SAGFF120A;UAS:GFP;UAS:zBoTxBLC:GFP fish. **a** Dorsal views of the brains from eight SAGFF120A;UAS:GFP (~10 months old) fish and eight SAGFF120A;UAS:GFP;UAS:zBoTxBLC:GFP (~10 months old) fish are shown. Scale bars: 1 mm. **b** Areas having more intensity than background (the maximum intensity measured in the posterior part of the telencephalon) were identified by using ImageJ [57] and shown in red. Scale bars: 500 μm. **c** Immunohistochemistry using anti-GFP (green) and anti-NeuN (a neuronal marker, magenta) of coronal sections of the telencephalon and hypothalamus of brain samples from these transgenic fish. The fish numbers correspond to the numbers of the dorsal view images. Scale bars, 200 μm. (PPTX 6544 kb)
