# Supplementary material for: Identification of a neuronal population in the telencephalon essential for fear conditioning in zebrafish
Source: BMC Biol. 2018 Apr 25;16:45. doi: 10.1186/s12915-018-0502-y (PMC5978991; doi:10.1186/s12915-018-0502-y)
Supplement: Supplementary file 5 — Figure S1. Performance of two-way active avoidance response of double transgenic (Gal4FF;UAS:zBoTxBLC:GFP) fish. The following Gal4FF transgenic fish lines were crossed with UAS:zBoTxBLC:GFP effector fish, and analyzed for two-way active avoidance fear conditioning. a hspGGFF10C (n = 6), b hspGGFF20A (n = 10), c hspGFF38B (n = 10), d hspGFF55B (n = 6), e SAGFF81B (n = 12), f SAGFF226F(n = 9), g SAGFF233A (n = 9), h SAGFF234A (n = 11), i hspGFFDMC12A (n = 12), j hspGFFDMC56B (n = 10), k hspGGFF19B (n = 9), l hspGGFF19C (n = 9), m hspGFF62A (n = 5), n gata6SAGFF94A (n = 6), o SAGFF27C (n = 6), p SAGFF38A (n = 8), q SAGFF87C (n = 5), r SAGFF92A (n = 8), s SAGFF183A (n = 5), t SAGFF195A (n = 6), u SAGFF212C (n = 5), v SAIGFF170B (n = 10), w hspGFFDMC76A (n = 10), x hspGFFDMC85C (n = 11). Mean ± SEM and avoidance (%) for individual fish are plotted. Performance of wild type fish (n = 28) described in Fig. 2 is shown in dotted lines. Two-way ANOVA, fish groups (wild type fish treated by CS-US, wild type fish treated by CS only and double transgenic fish including fish described in Figs. 2 and 3 × training session days (day 1, day 5), was performed (F = 7.236, P < 0.0001). Dunnett’s multiple comparison post-hoc tests were performed between avoidance percentage of wild type fish and double transgenic fish on session day 1 and day 5. *P < 0.05, **P < 0.01, ***P < 0.001, ****P < 0.0001; ns, not significant (P > 0.05). a–j Reduced performance of the active avoidance response was observed. k–x Performance of the active avoidance response was not significantly different between wild type and the double transgenic fish. (PPTX 8767 kb) [file 12915_2018_502_MOESM2_ESM.pptx]

## Slide 1
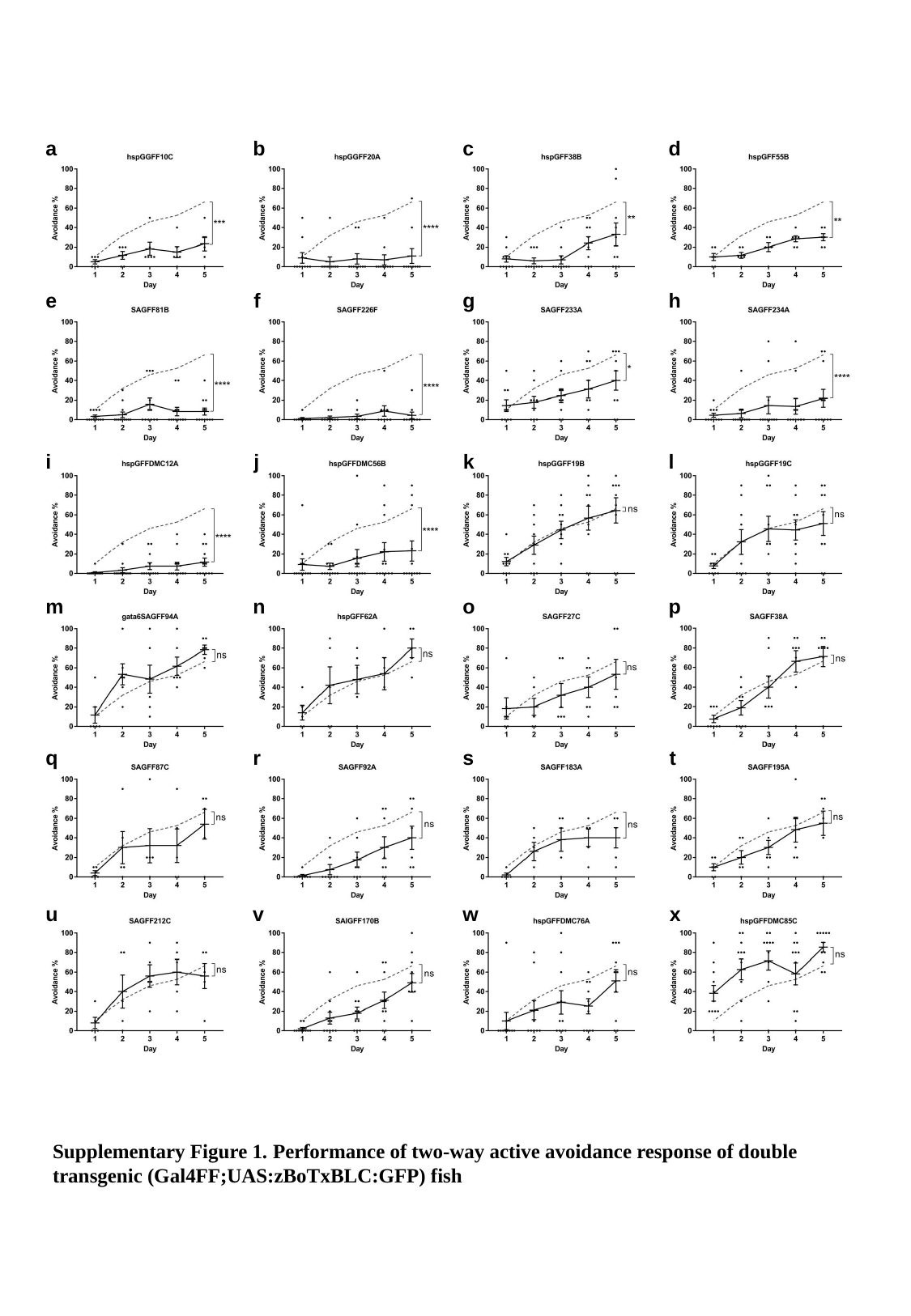

a
b
c
d
e
f
g
h
i
j
k
l
m
n
o
p
q
r
s
t
u
v
w
x
Supplementary Figure 1. Performance of two-way active avoidance response of double transgenic (Gal4FF;UAS:zBoTxBLC:GFP) fish
