# Supplementary material for: Identification of a neuronal population in the telencephalon essential for fear conditioning in zebrafish
Source: BMC Biol. 2018 Apr 25;16:45. doi: 10.1186/s12915-018-0502-y (PMC5978991; doi:10.1186/s12915-018-0502-y)
Supplement: Supplementary file 6 — Figure S2. GFP expression patterns of 16 Gal4FF;UAS:GFP fish that showed reduced performance of the active avoidance response. A dorsal view, a ventral view, and a schematic side view with positions of coronal sections are shown on the top. Serial coronal sections with position numbers are shown in the bottom. a hspGGFF10C, b hspGGFF20A, c hspGFF38B, d hspGFF55B, e SAGFF36B, f SAGFF70A, g SAGFF81B, h SAGFF120A, i SAGFF226F, j SAGFF228A, k SAGFF231A, l SAGFF233A, m SAGFF234A, n SAGFF234D, o hspGFFDMC12A, p hspGFFDMC56B. Scale bars in whole brain images: 500 μm. Scale bars in coronal section images: 200 μm. (PDF 3264 kb) [file 12915_2018_502_MOESM3_ESM.pdf]

Supplementary Figure 2. GFP expression patterns of 16 Gal4FF;UAS:GFP fish that showed reduced performance of the active avoidance response

a

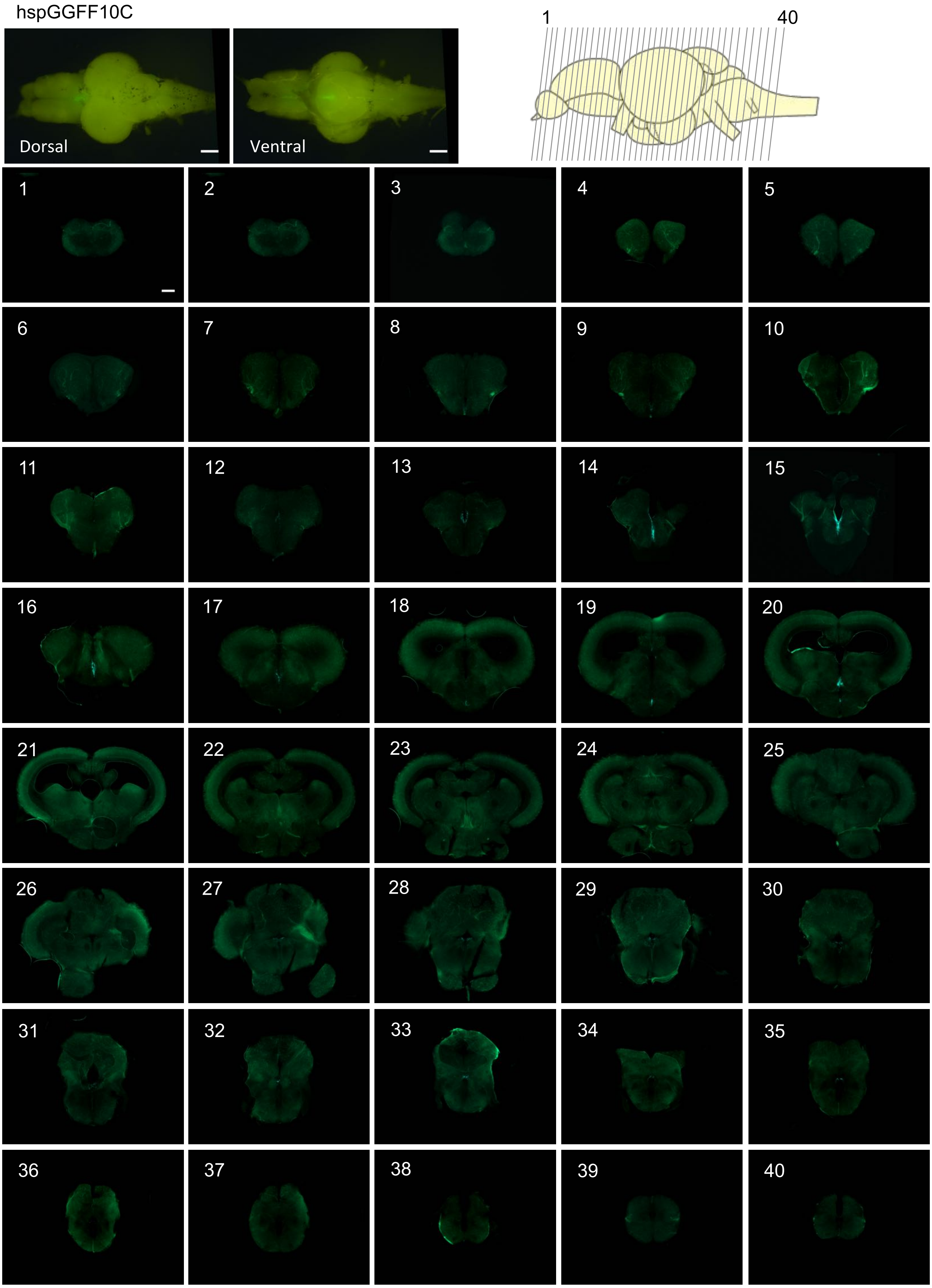
hspGGFF10C
Dorsal
Ventral
1
40
1
2
3
4
5
6
7
8
9
10
11
12
13
14
15
16
17
18
19
20
21
22
23
24
25
26
27
28
29
30
31
32
33
34
35
36
37
38
39
40

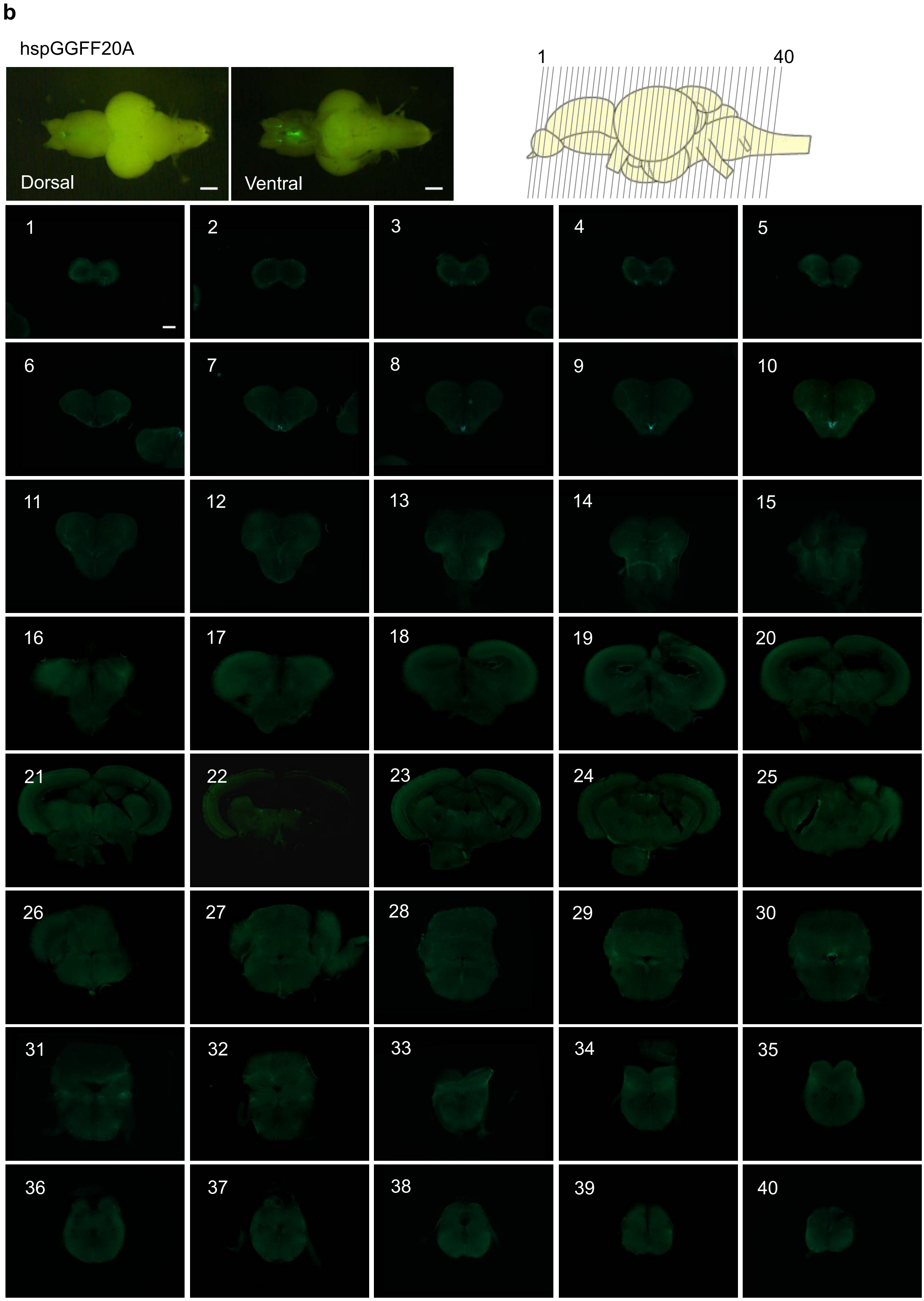
b
hspGGFF20A
Dorsal
Ventral
1
40
1
2
3
4
5
6
7
8
9
10
11
12
13
14
15
16
17
18
19
20
21
22
23
24
25
26
27
28
29
30
31
32
33
34
35
36
37
38
39
40

c

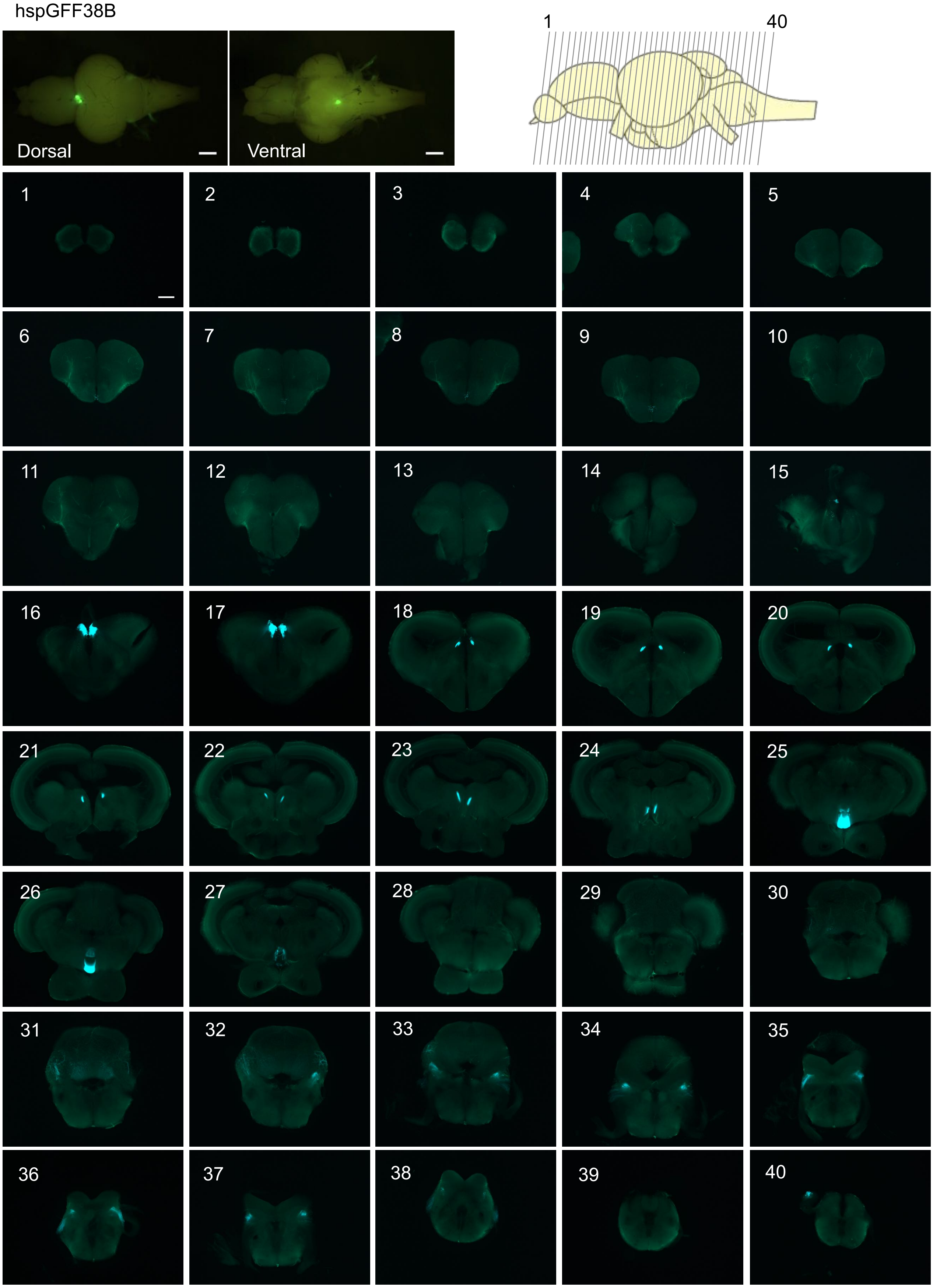
hspGFF38B
Dorsal
Ventral
1
40
1
2
3
4
5
6
7
8
9
10
11
12
13
14
15
16
17
18
19
20
21
22
23
24
25
26
27
28
29
30
31
32
33
34
35
36
37
38
39
40

d

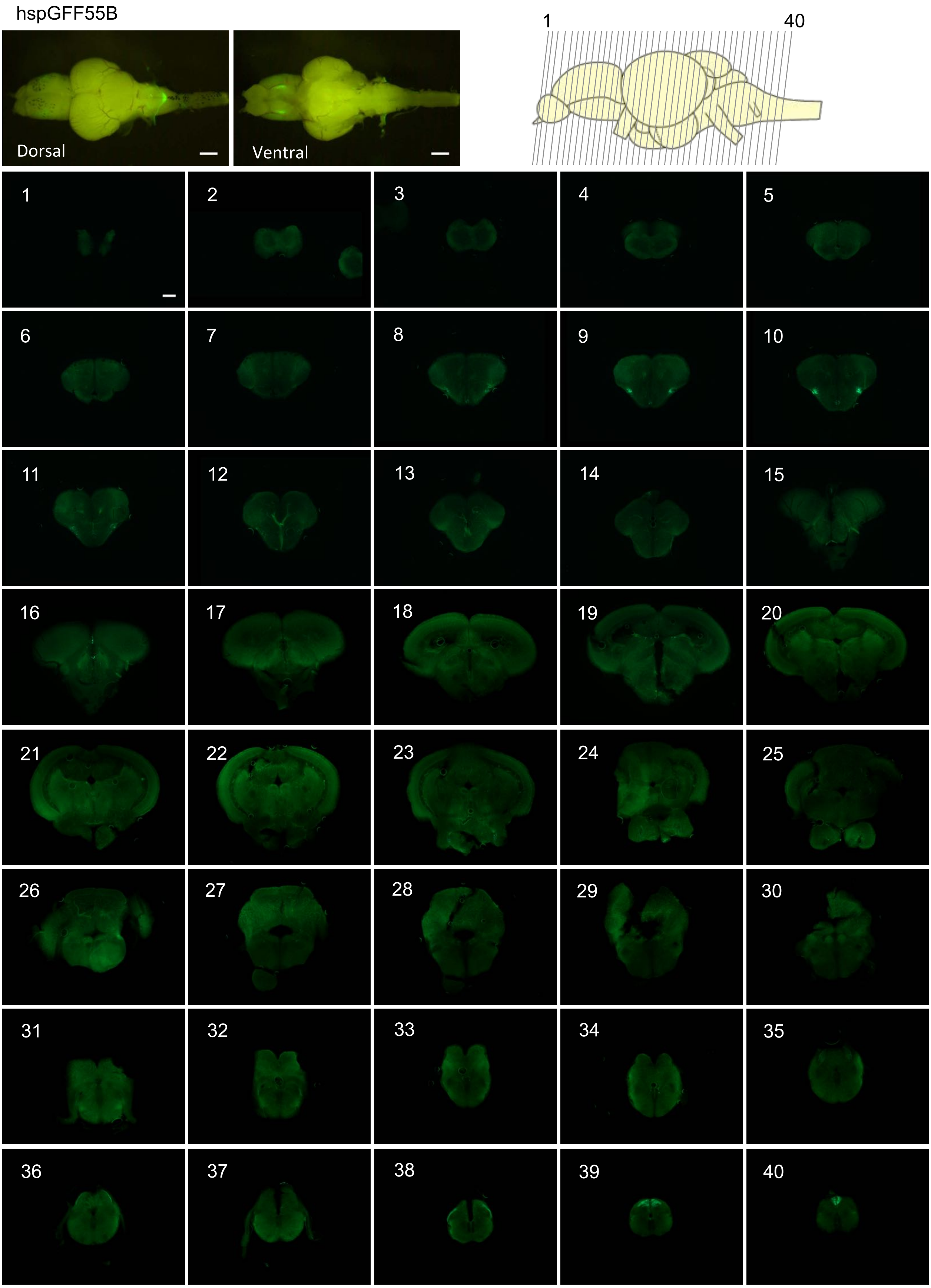
hspGFF55B
Dorsal
Ventral
1
40
1
2
3
4
5
6
7
8
9
10
11
12
13
14
15
16
17
18
19
20
21
22
23
24
25
26
27
28
29
30
31
32
33
34
35
36
37
38
39
40

e

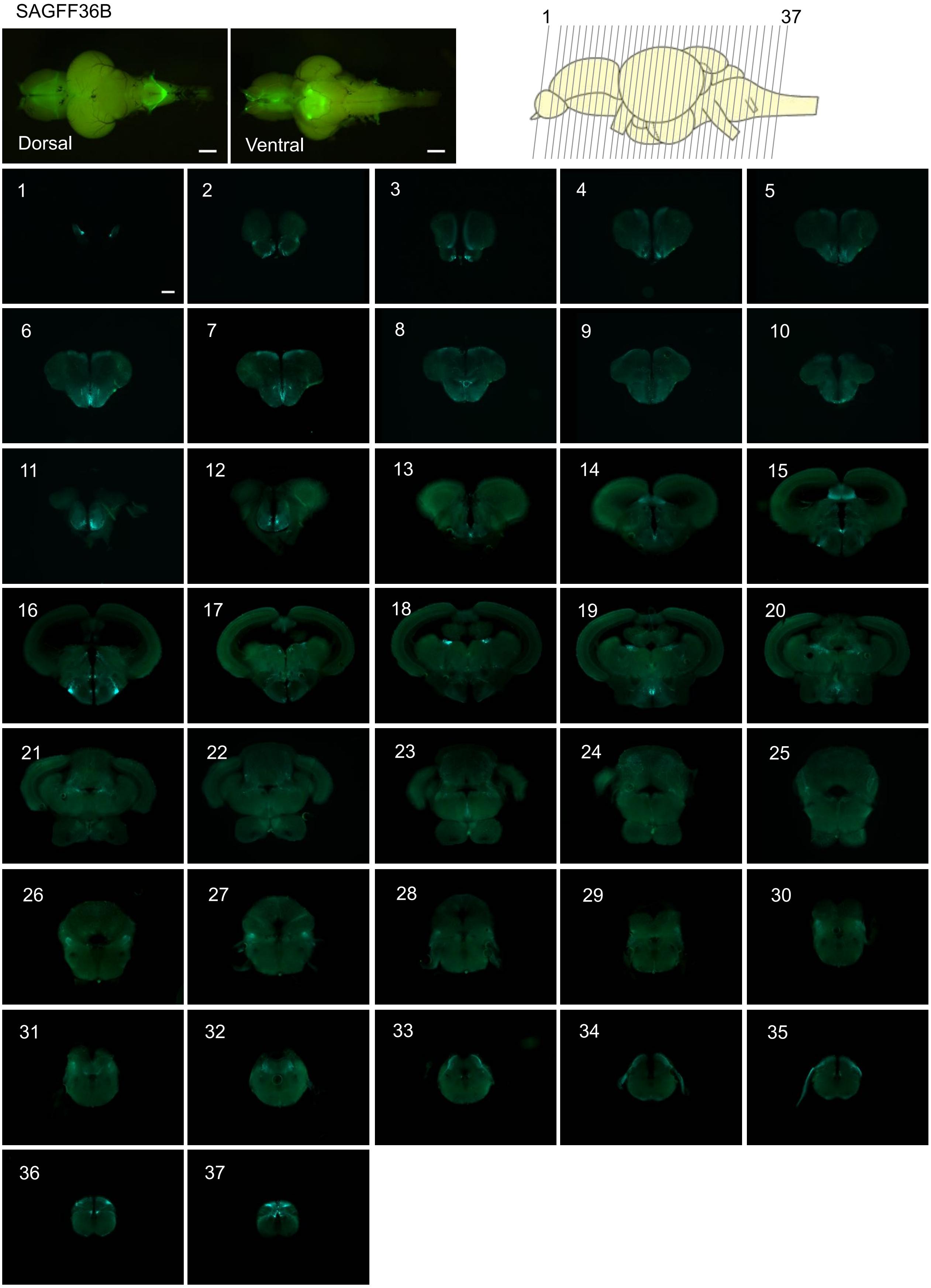
SAGFF36B
Dorsal
Ventral
1
37
1
2
3
4
5
6
7
8
9
10
11
12
13
14
15
16
17
18
19
20
21
22
23
24
25
26
27
28
29
30
31
32
33
34
35
36
37

f

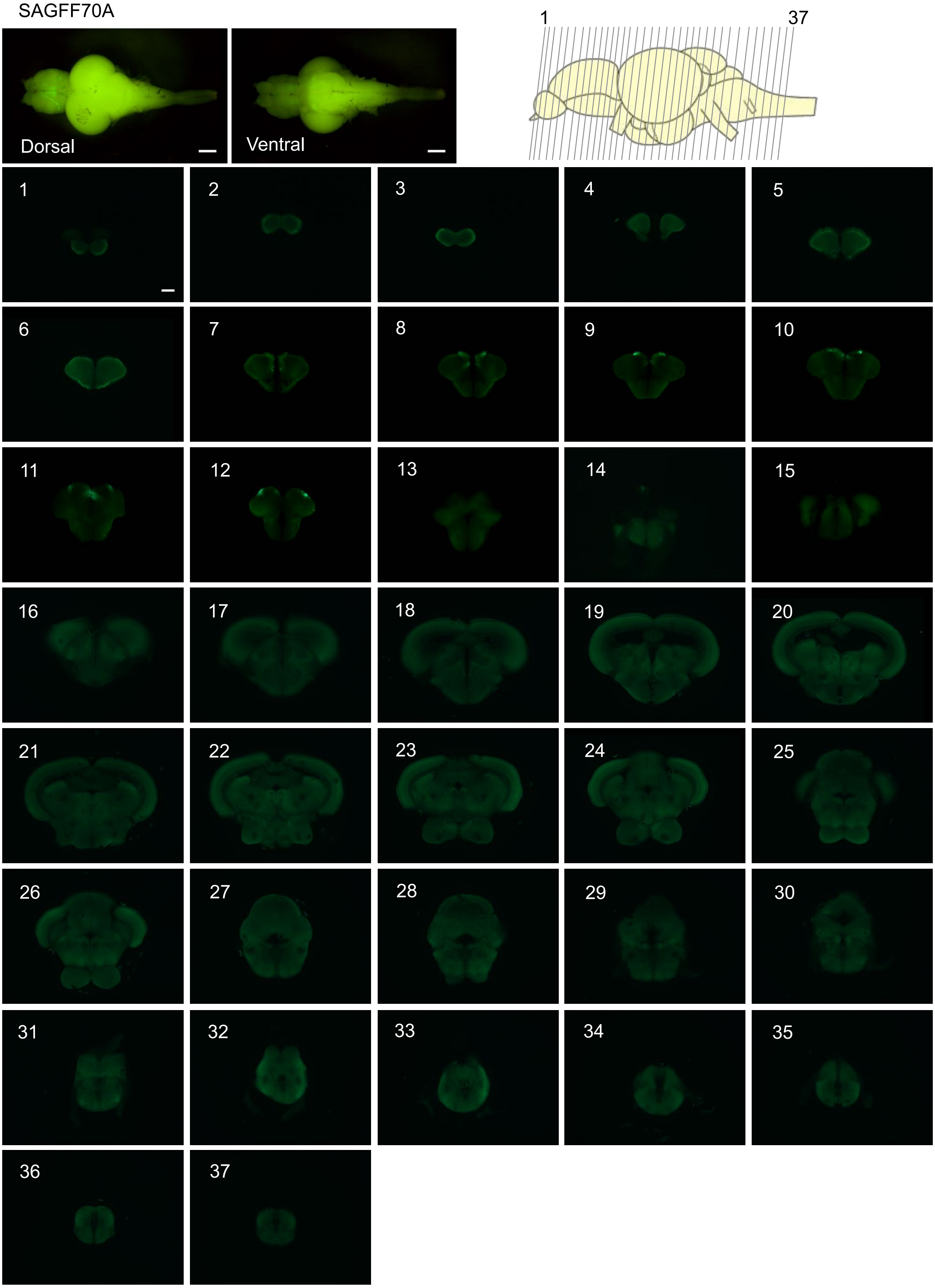
SAGFF70A
Dorsal
Ventral
1
37
1
2
3
4
5
6
7
8
9
10
11
12
13
14
15
16
17
18
19
20
21
22
23
24
25
26
27
28
29
30
31
32
33
34
35
36
37

g

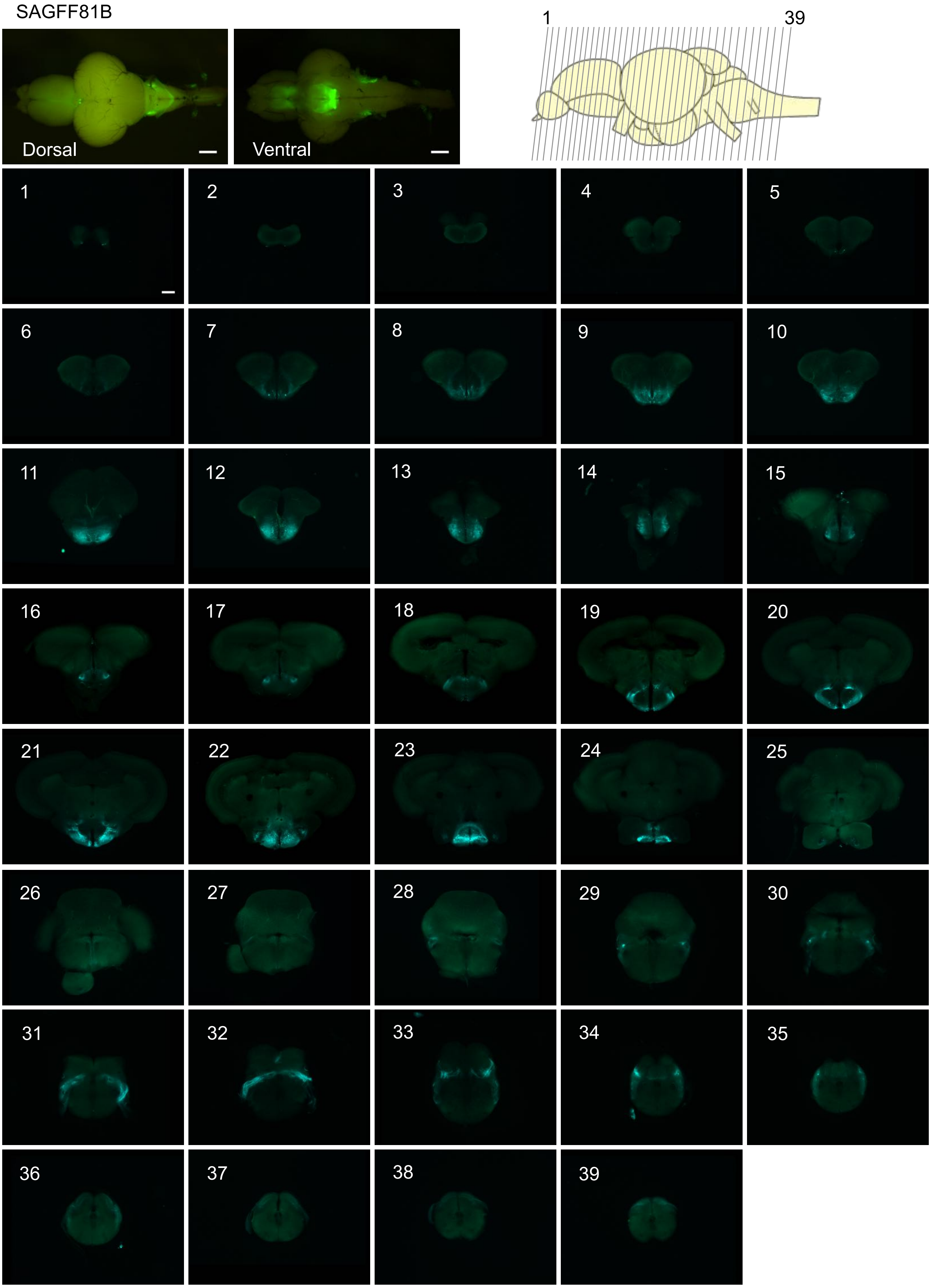
SAGFF81B
Dorsal
Ventral
1
39
1
2
3
4
5
6
7
8
9
10
11
12
13
14
15
16
17
18
19
20
21
22
23
24
25
26
27
28
29
30
31
32
33
34
35
36
37
38
39

h

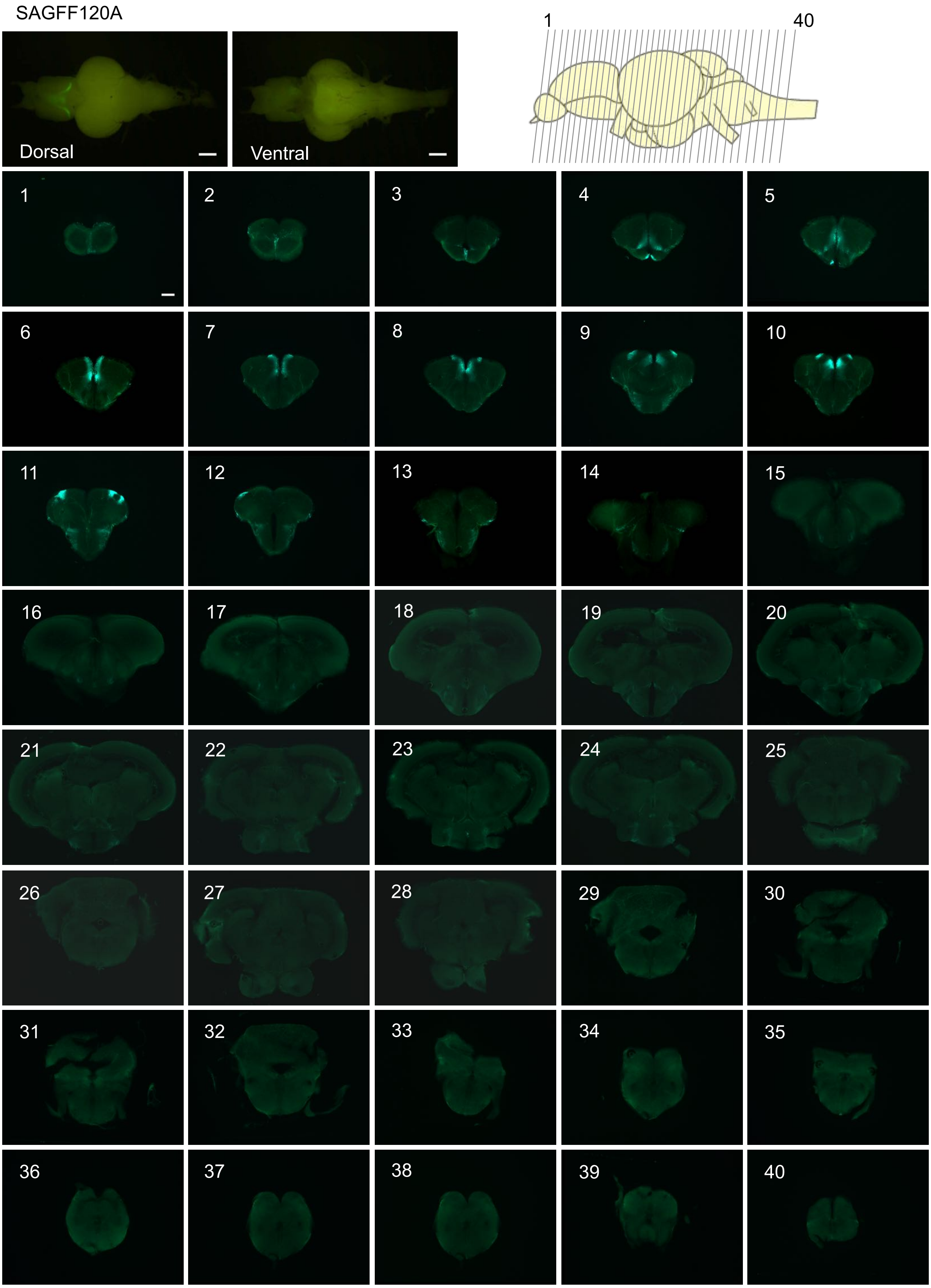
SAGFF120A
Dorsal
Ventral
1
40
1
2
3
4
5
6
7
8
9
10
11
12
13
14
15
16
17
18
19
20
21
22
23
24
25
26
27
28
29
30
31
32
33
34
35
36
37
38
39
40

i

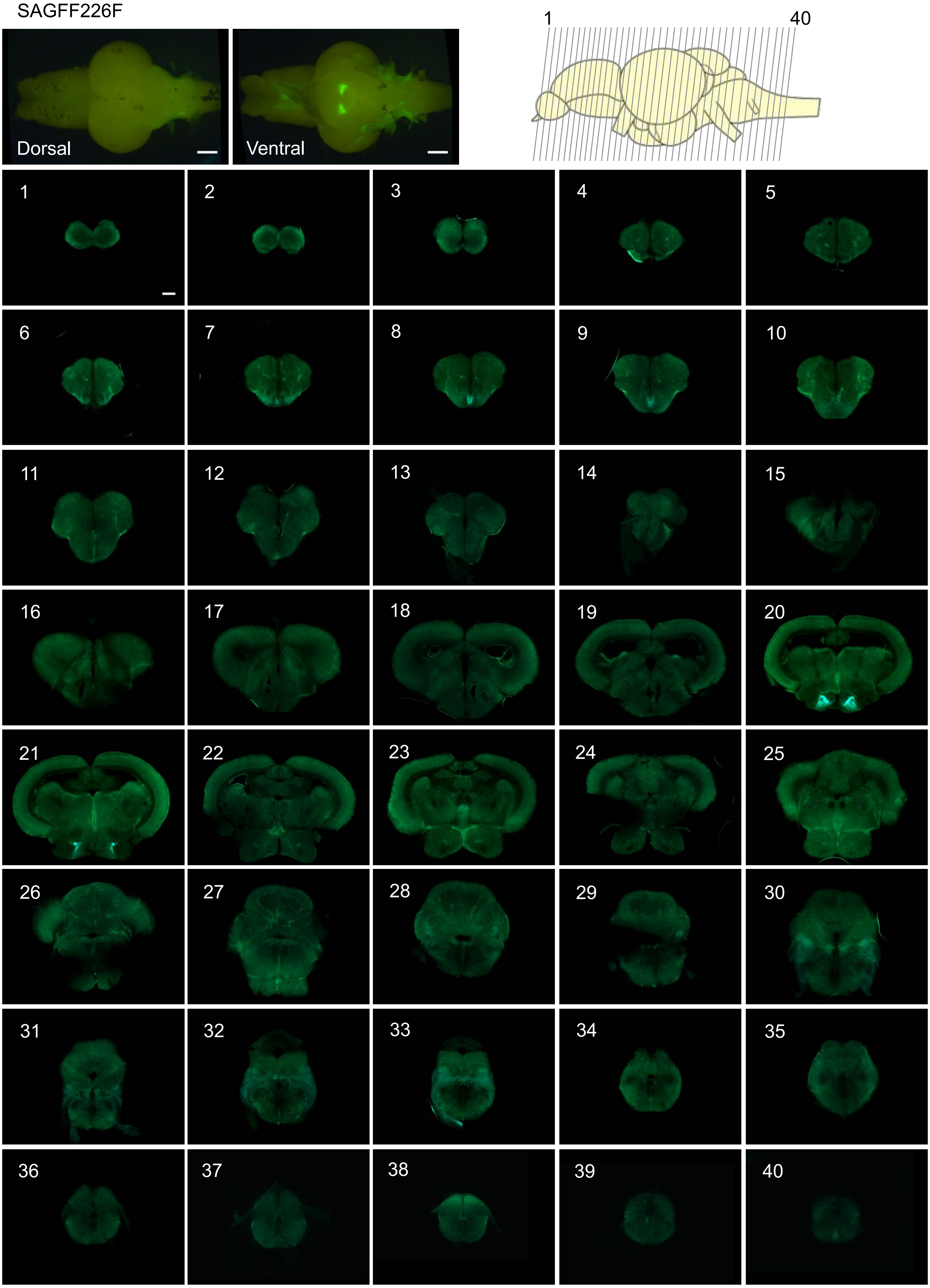
SAGFF226F
Dorsal
Ventral
1
40
1
2
3
4
5
6
7
8
9
10
11
12
13
14
15
16
17
18
19
20
21
22
23
24
25
26
27
28
29
30
31
32
33
34
35
36
37
38
39
40

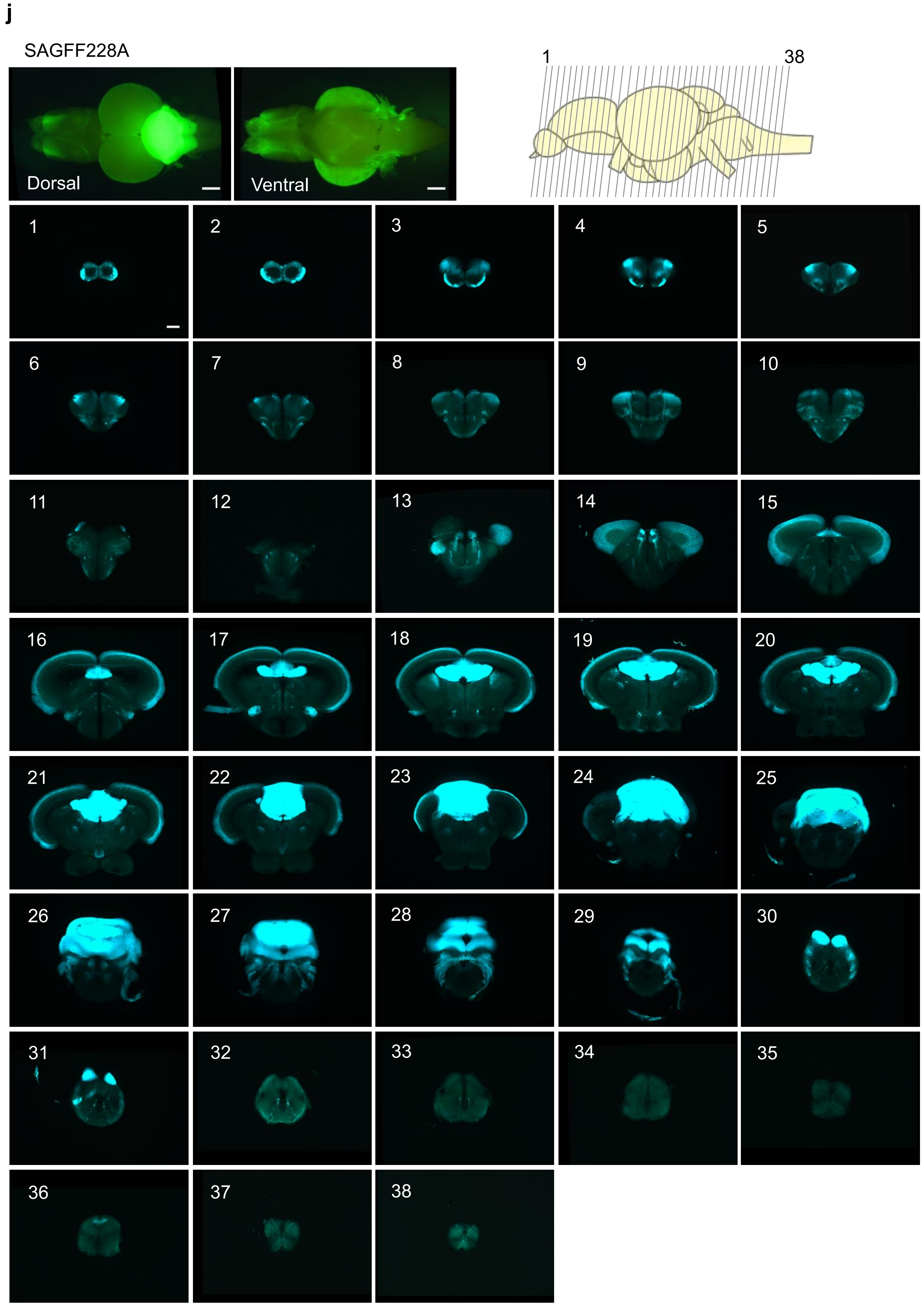
j
SAGFF228A
Dorsal
Ventral
1
38
1
2
3
4
5
6
7
8
9
10
11
12
13
14
15
16
17
18
19
20
21
22
23
24
25
26
27
28
29
30
31
32
33
34
35
36
37
38

k

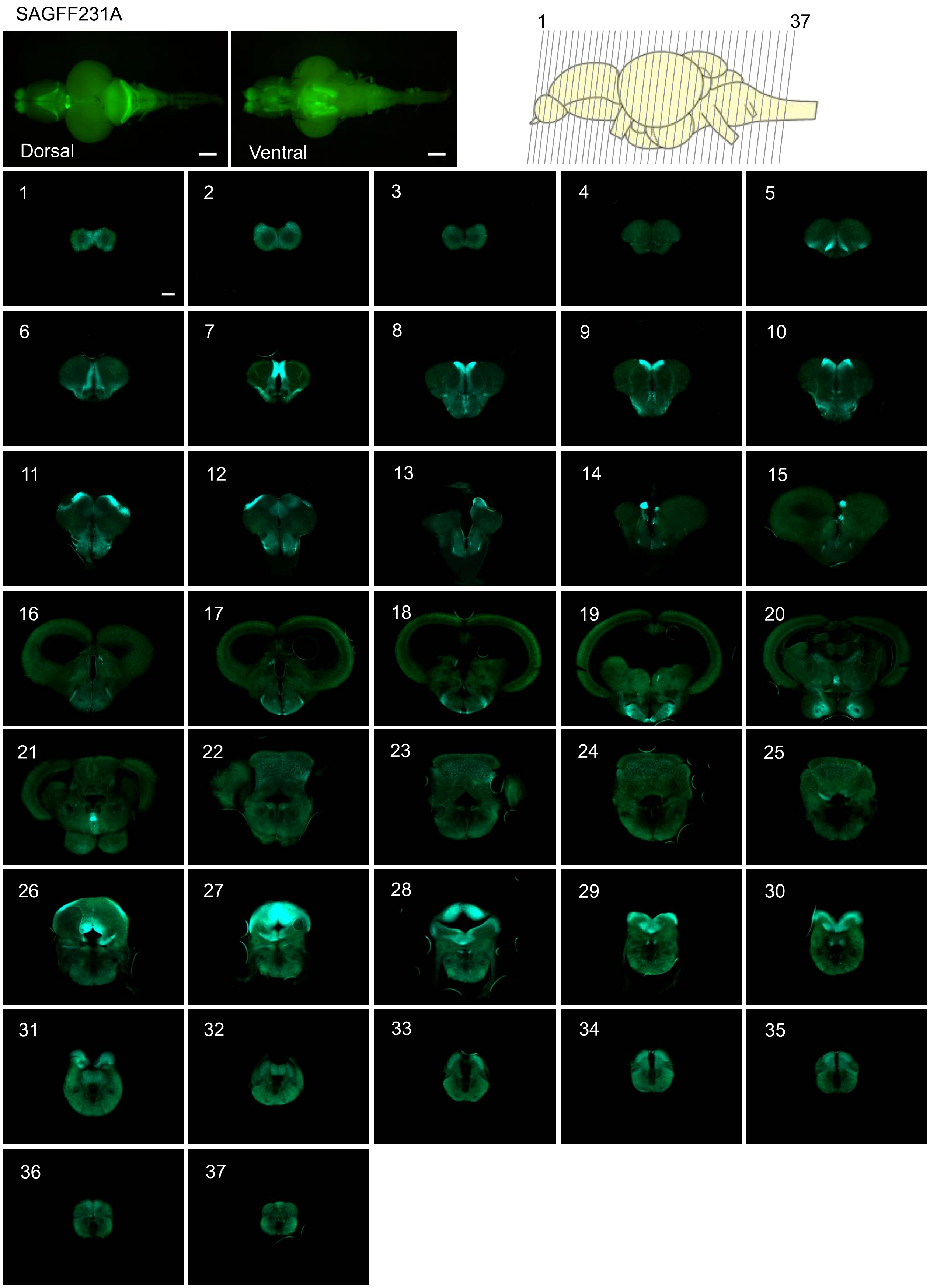
SAGFF231A
Dorsal
Ventral
1
37
1
2
3
4
5
6
7
8
9
10
11
12
13
14
15
16
17
18
19
20
21
22
23
24
25
26
27
28
29
30
31
32
33
34
35
36
37

I

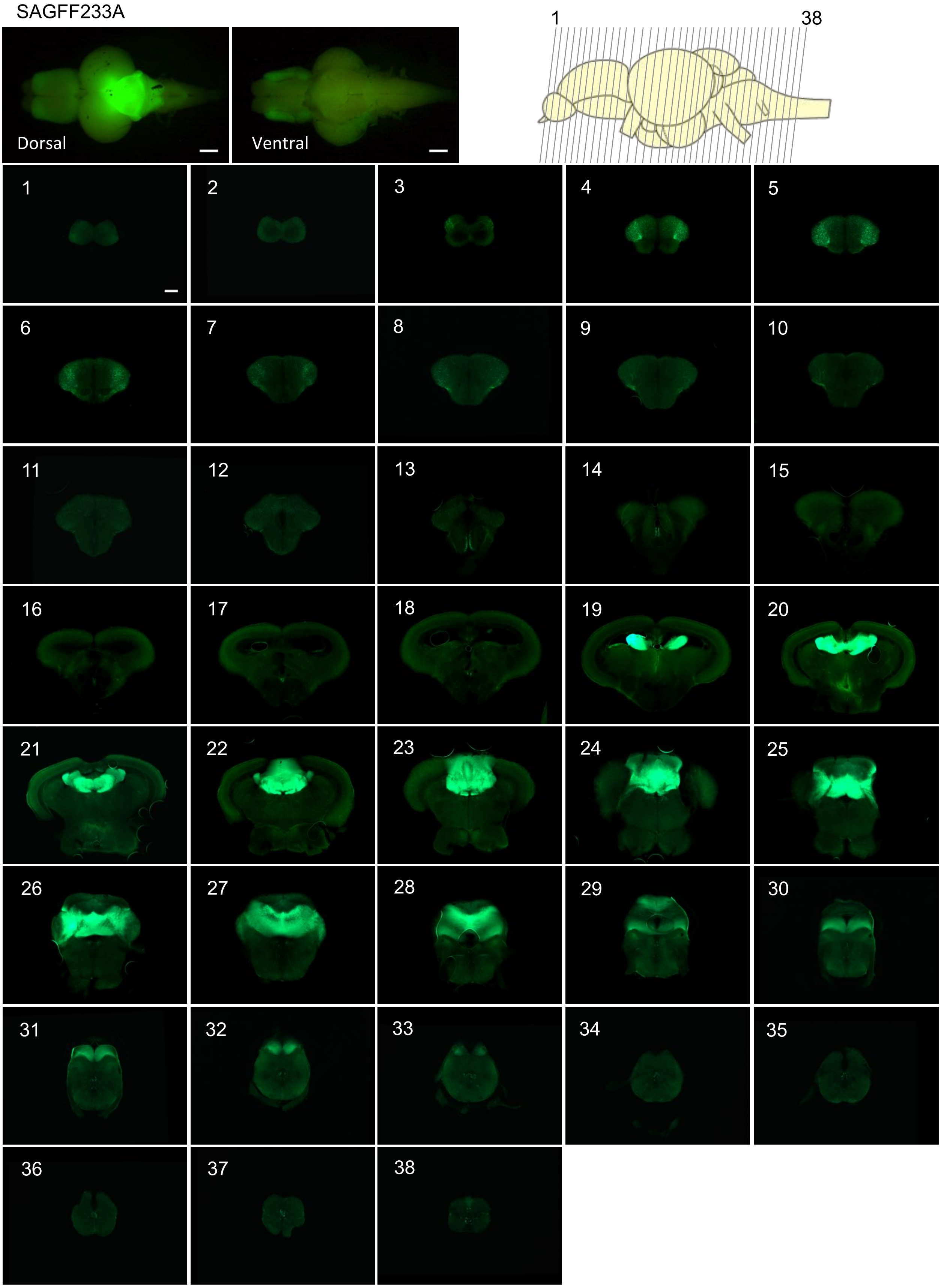
SAGFF233A
Dorsal
Ventral
1
38
1
2
3
4
5
6
7
8
9
10
11
12
13
14
15
16
17
18
19
20
21
22
23
24
25
26
27
28
29
30
31
32
33
34
35
36
37
38

m

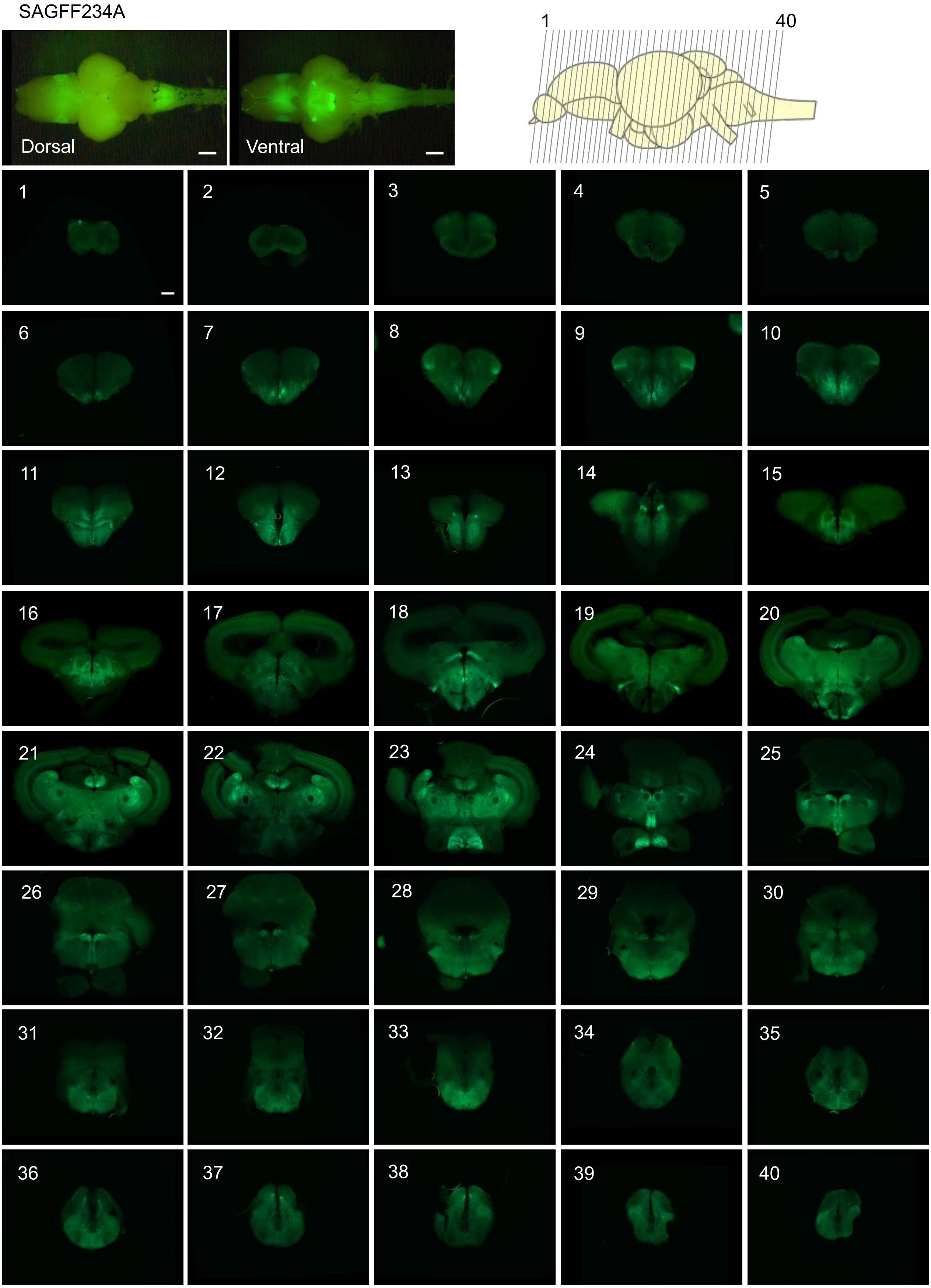
SAGFF234A
Dorsal
Ventral
1
40
1
2
3
4
5
6
7
8
9
10
11
12
13
14
15
16
17
18
19
20
21
22
23
24
25
26
27
28
29
30
31
32
33
34
35
36
37
38
39
40

n

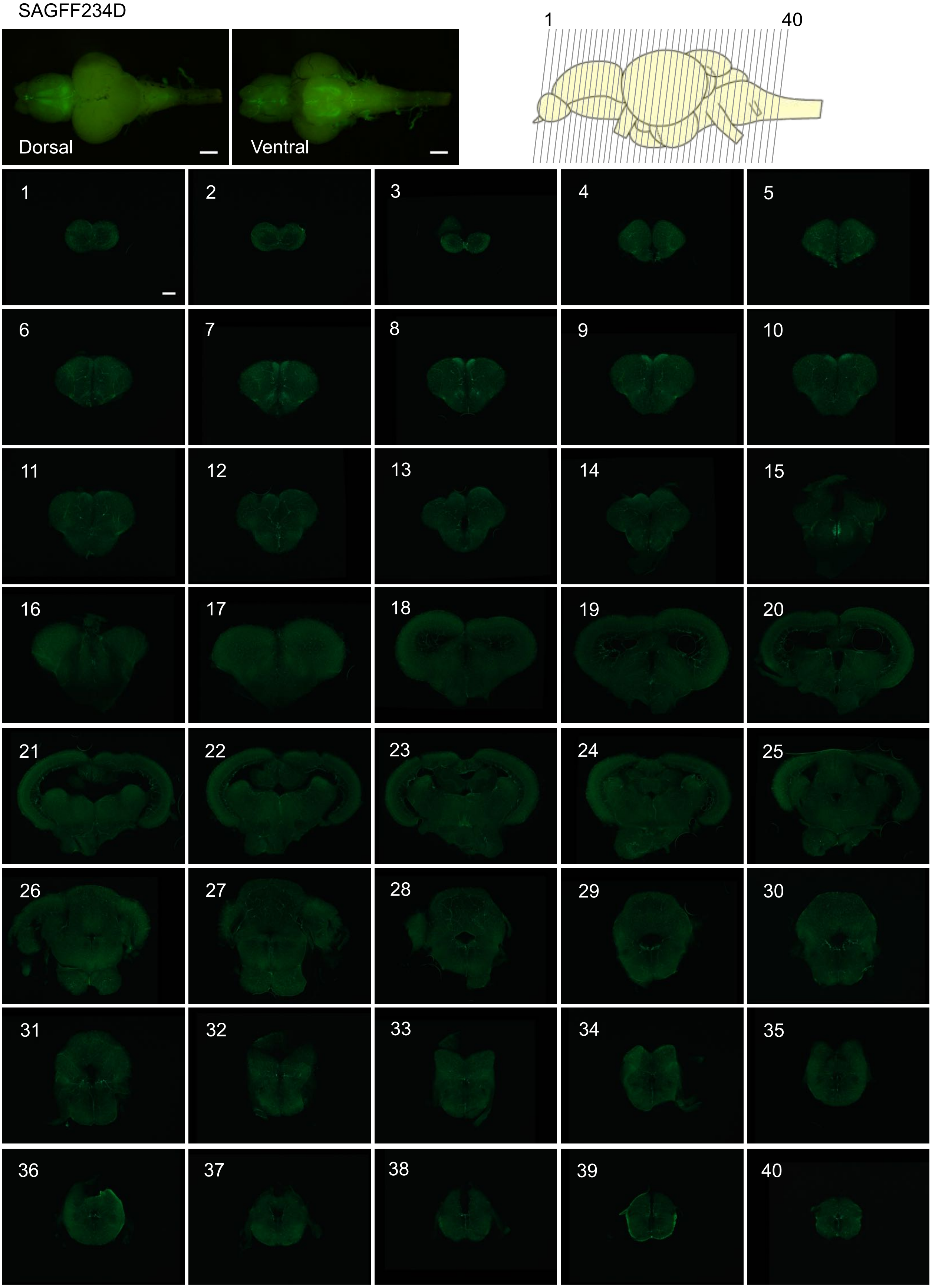
SAGFF234D
Dorsal
Ventral
1
40
1
2
3
4
5
6
7
8
9
10
11
12
13
14
15
16
17
18
19
20
21
22
23
24
25
26
27
28
29
30
31
32
33
34
35
36
37
38
39
40

O

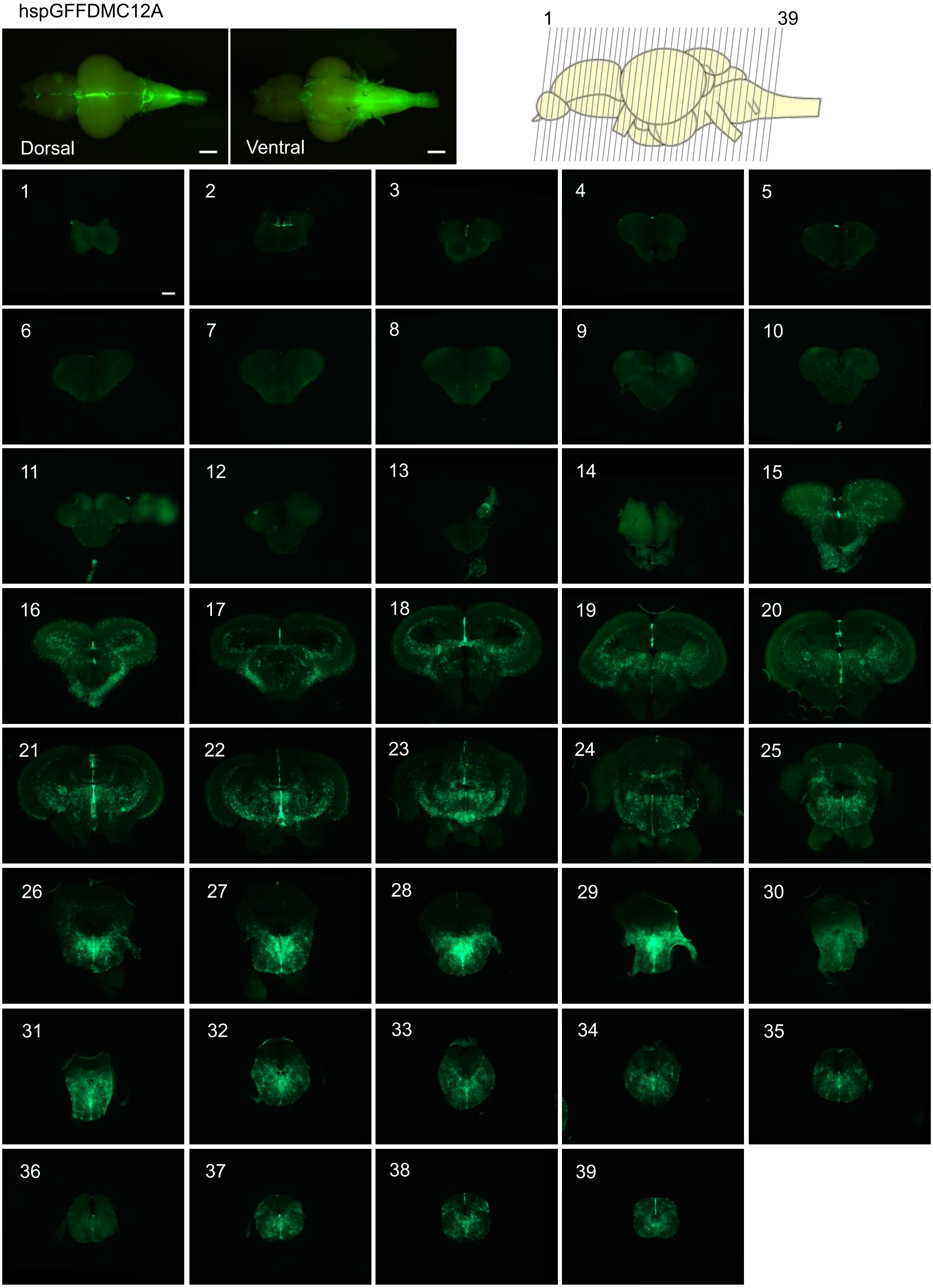
hspGFFDMC12A
Dorsal
Ventral
1
39
1
2
3
4
5
6
7
8
9
10
11
12
13
14
15
16
17
18
19
20
21
22
23
24
25
26
27
28
29
30
31
32
33
34
35
36
37
38
39

p

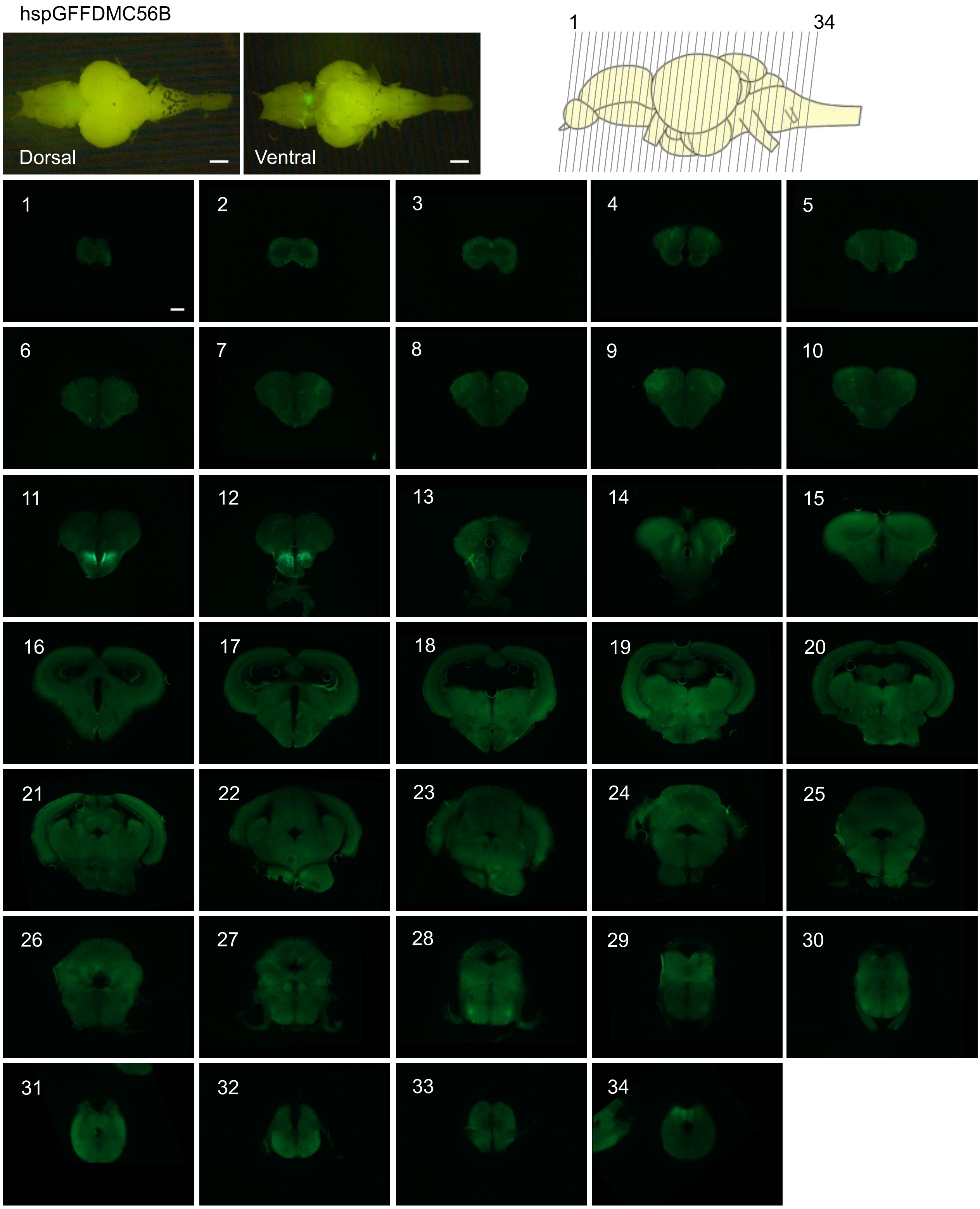
hspGFFDMC56B
Dorsal
Ventral
1
34
1
2
3
4
5
6
7
8
9
10
11
12
13
14
15
16
17
18
19
20
21
22
23
24
25
26
27
28
29
30
31
32
33
34
