# Supplementary material for: Identification of a neuronal population in the telencephalon essential for fear conditioning in zebrafish
Source: BMC Biol. 2018 Apr 25;16:45. doi: 10.1186/s12915-018-0502-y (PMC5978991; doi:10.1186/s12915-018-0502-y)
Supplement: Supplementary file 7 — Figure S3. GFP expression patterns in SAGFF120A;UAS:GFP fish at embryonic stages. Bright field and fluorescent images of frontal and lateral views of SAGFF120A;UAS:GFP fish at 24, 48, 72, and 96 hpf. Scale bar, 200 mm. (PPTX 1417 kb) [file 12915_2018_502_MOESM4_ESM.pptx]

## Slide 1
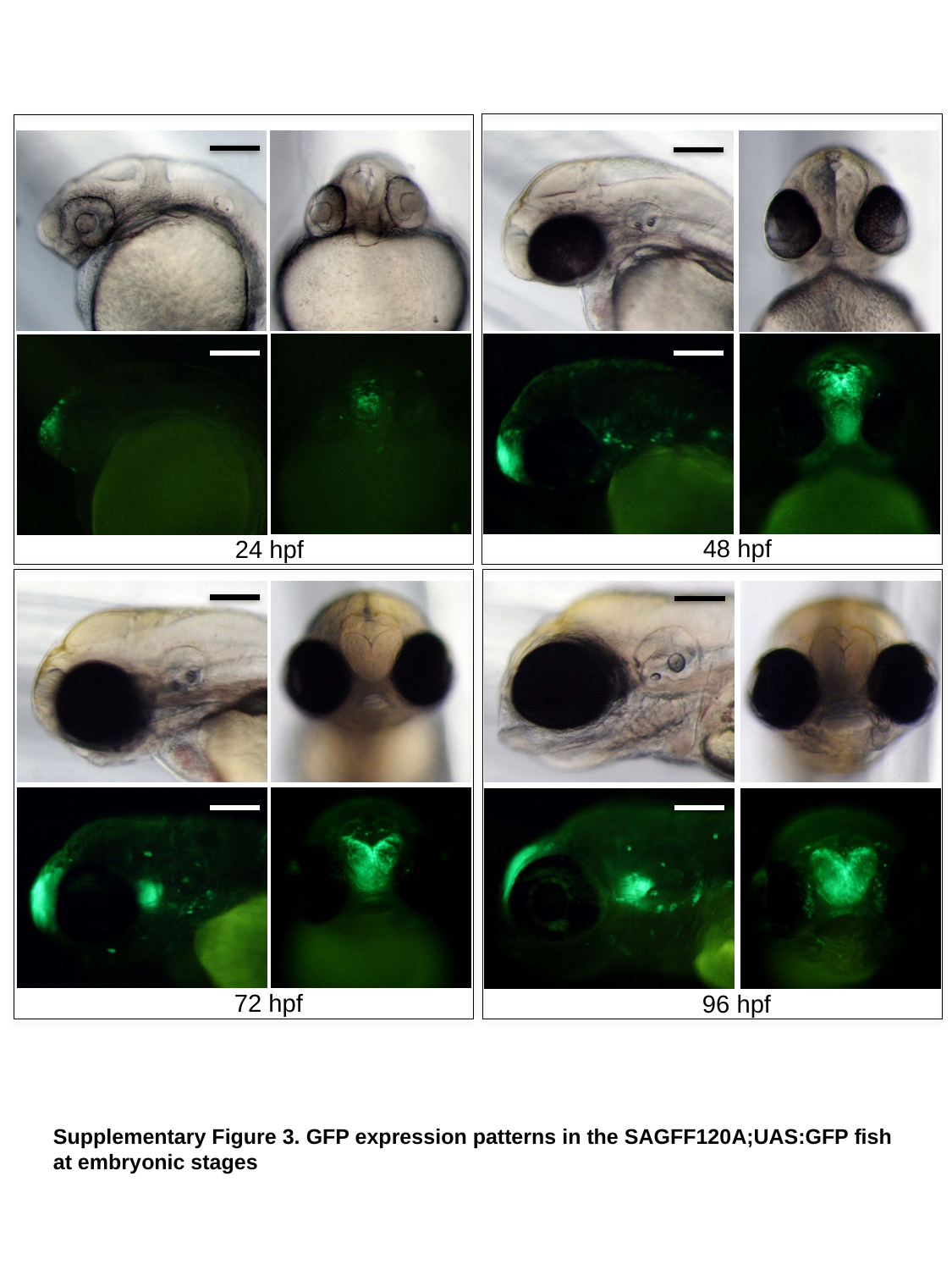

48 hpf
24 hpf
72 hpf
96 hpf
Supplementary Figure 3. GFP expression patterns in the SAGFF120A;UAS:GFP fish at embryonic stages
