# Supplementary material for: Identification of a neuronal population in the telencephalon essential for fear conditioning in zebrafish
Source: BMC Biol. 2018 Apr 25;16:45. doi: 10.1186/s12915-018-0502-y (PMC5978991; doi:10.1186/s12915-018-0502-y)
Supplement: Supplementary file 8 — Figure S4. GFP expression patterns in SAGFF120A;UAS:GFP and SAGFF120A;UAS:GFP;UAS:zBoTxBLC:GFP fish. a Dorsal views of the brains from eight SAGFF120A;UAS:GFP (~10 months old) fish and eight SAGFF120A;UAS:GFP;UAS:zBoTxBLC:GFP (~10 months old) fish are shown. Scale bars: 1 mm. b Areas having more intensity than background (the maximum intensity measured in the posterior part of the telencephalon) were identified by using ImageJ [57] and shown in red. Scale bars: 500 μm. c Immunohistochemistry using anti-GFP (green) and anti-NeuN (a neuronal marker, magenta) of coronal sections of the telencephalon and hypothalamus of brain samples from these transgenic fish. The fish numbers correspond to the numbers of the dorsal view images. Scale bars, 200 μm. (PPTX 6544 kb) [file 12915_2018_502_MOESM8_ESM.pptx]

## Slide 1
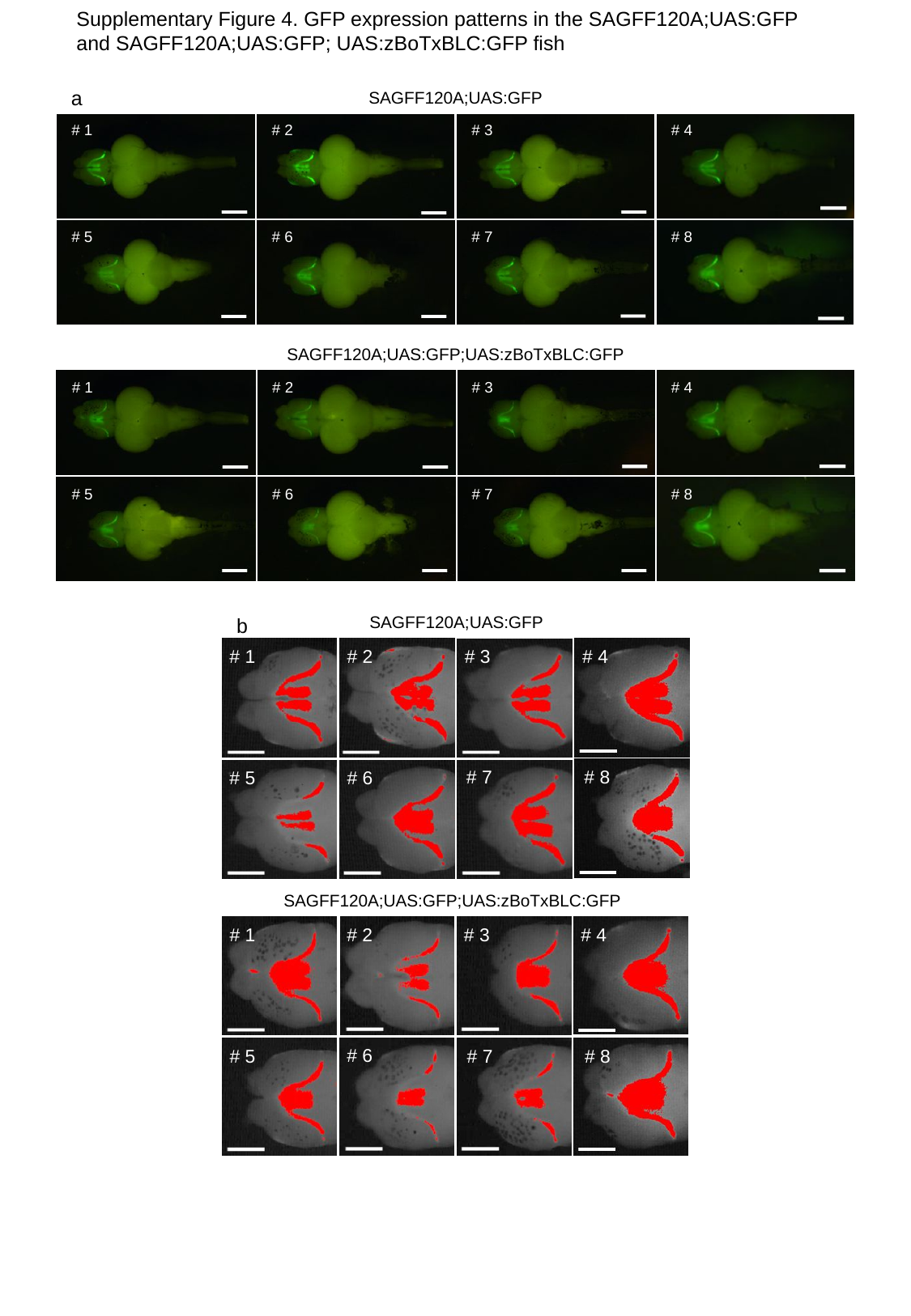

Supplementary Figure 4. GFP expression patterns in the SAGFF120A;UAS:GFP and SAGFF120A;UAS:GFP; UAS:zBoTxBLC:GFP fish
a
SAGFF120A;UAS:GFP
# 4
# 3
# 1
# 2
# 8
# 5
# 6
# 7
SAGFF120A;UAS:GFP;UAS:zBoTxBLC:GFP
# 4
# 3
# 1
# 2
# 8
# 5
# 6
# 7
SAGFF120A;UAS:GFP
b
# 4
# 1
# 2
# 3
# 7
# 8
# 5
# 6
SAGFF120A;UAS:GFP;UAS:zBoTxBLC:GFP
# 1
# 2
# 3
# 4
# 6
# 5
# 7
# 8

## Slide 2
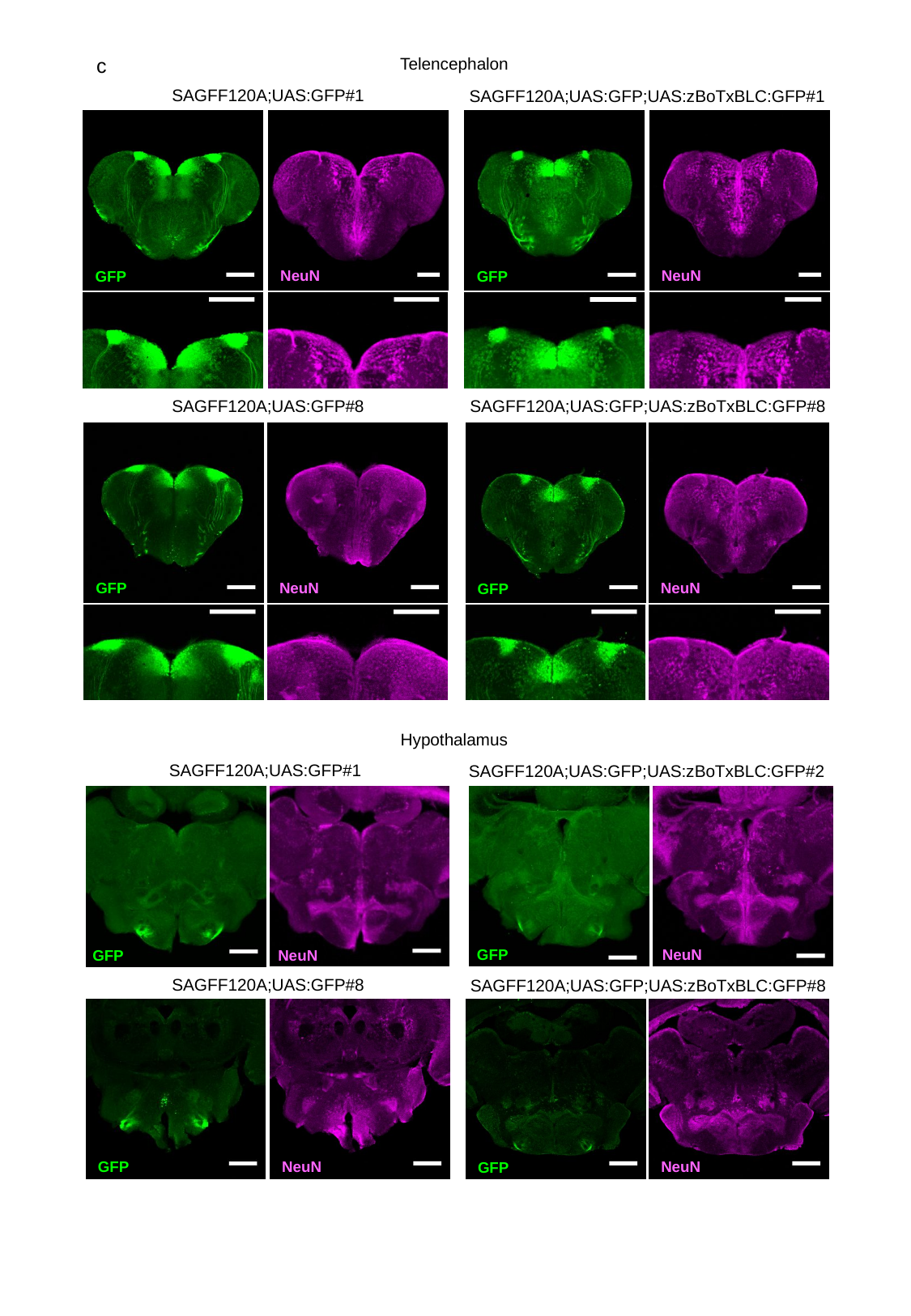

Telencephalon
c
SAGFF120A;UAS:GFP#1
SAGFF120A;UAS:GFP;UAS:zBoTxBLC:GFP#1
NeuN
NeuN
GFP
GFP
SAGFF120A;UAS:GFP#8
SAGFF120A;UAS:GFP;UAS:zBoTxBLC:GFP#8
NeuN
NeuN
GFP
GFP
Hypothalamus
SAGFF120A;UAS:GFP#1
SAGFF120A;UAS:GFP;UAS:zBoTxBLC:GFP#2
GFP
NeuN
GFP
NeuN
SAGFF120A;UAS:GFP#8
SAGFF120A;UAS:GFP;UAS:zBoTxBLC:GFP#8
NeuN
NeuN
GFP
GFP
